# Now that I see it your way, I choose you: Visuo-spatial perspective-taking affects partner selection during coalition formation

**DOI:** 10.1177/17470218251358231

**Published:** 2025-07-08

**Authors:** Anabela Cantiani, Ilja van Beest, Thorsten M Erle

**Affiliations:** Department of Social Psychology, Tilburg School of Social and Behavioral Sciences, Tilburg University, Tilburg, The Netherlands

**Keywords:** (Visuo-spatial) perspective-taking, partner selection, coalition formation, social cognition, embodiment

## Abstract

Humans constantly form coalitions to achieve shared goals, and current theories of coalition formation assume that this process is solely guided by economic incentives. However, this assumption neglects the importance of psychological processes that contribute to coalition formation, which is especially problematic in scenarios where the economic motives of potential partners are (initially) indistinguishable. This research investigates the impact of one psychological process, visuo-spatial perspective-taking (VSPT), on coalition formation. We hypothesized that adopting the perspective of a potential coalition partner increases the likelihood of forming a coalition with them, compared to partners viewed from an egocentric standpoint. Importantly, this is not because this person is economically more advantageous, but because perspective-taking increases liking for and similarity to others. These effects, however, stem from an embodied simulation of physical closeness that only some participants (embodiers) but not others (disembodiers) engage in, suggesting a moderation of this preference by perspective-taking strategy. Across five experiments (*N* = 2,340), participants completed a VSPT task before engaging in a hypothetical coalition formation negotiation with the targets presented during the VSPT task. Meta-analytically, our data suggests that embodiers indeed showed increased liking and similarity after perspective-taking, while disembodiers did not. However, unexpectedly, not only embodiers but also disembodiers selected partners whose perspective they took more often as a coalition partner. We discuss potential explanations for this preference in disembodiers, implications of our work for theories of coalition formation, and for research on different strategies for perspective-taking.

Human societies are *ultrasocial* because numerous desired outcomes are only attainable by pooling resources with other individuals or groups (Campbell, 1983; Henrich & Muthukrishna, 2021), or put differently: by forming coalitions. Today, coalitions are formed everywhere, for example, when friends collaborate on a community clean-up initiative (Clean Up The World, n.d.), when project developers partner with various stakeholders to acquire land for development, or when nations or political parties unite in (global) initiatives to address war or other large-scale societal problems (Stop the War Coalition, n.d.).

In the complex societies of today, coalition formation scenarios often also involve multiple partners to cooperate with. In such situations, selecting the right partner is difficult but crucial because different parties bring conflicting interests to the table, creating a mixed-motive situation (Columbus et al., 2021). Sometimes, the motives of potential coalition partners can be explicit, but other times, people have very limited information about them. How then do individuals navigate these situations to select a coalition partner?

## Coalition partner selection

Partner selection in coalition formation scenarios has been extensively studied, especially using “simple weighted majority games” (for an overview, see Kahan & Rapoport, 1984). Grounded in game theory, predominant coalition theories posit that partner selection depends entirely on static features of the game scenario. For example, consider a weighted majority game where players possess different resources, like hectares of land or votes in an election. In a game with a 5(4–3–2) configuration, Player A has 4 resources, Player B has 3, and Player C has 2. The five signifies that a total of 5 units (i.e., any coalition between at least two players) is needed to form a winning coalition that gets to distribute an individually unattainable prize amongst its members. The main questions then are who will cooperate with whom, and how will the prize (e.g., money) will be allocated?

According to coalition theories, both partner selection and money allocation are predicted solely based on the static features of the game, such as the resources each player controls (e.g., minimum resource theory) and/or the number of coalitions they can potentially form (e.g., minimum power theory). In the example above, minimum resource theory (Gamson, 1961) predicts that Player C would expect Player A to use their resource advantage (4:2) to propose a proportional 2:1 split. However, Player B, with a closer ratio (3:2), might offer a more favorable 3:2 split, making Player B a more attractive partner for Player C, since Player C would receive 40% of the total prize with Player B, compared to 33% with Player A.

When clear, objective cues like resources are available, as in the 5(4–3–2) game, it is reasonable to assume that these cues guide partner selection. However, such scenarios are not typical in real-life coalition formation. In politics and business, the formation of a coalition is often preceded by extensive talks where “players” gauge each other’s intentions. These intentions are rarely static or easily visible, complicating the decision-making process.

However, in real-world situations where cues are less clear or absent, we know much less about how partner selection happens. For example, consider a 2(1–1–1) simple weighted majority game, where all players have equal resources (Van Beest et al., 2004). This configuration eliminates the economic factors that coalition theories typically rely on to predict partner preferences. In this case, coalition theories would predict random partner selection, as the static features of the game do not favor either partner over the other. Yet, we know from social psychological research that often negotiation-irrelevant cues, such as the likability of a person or their perceived similarity, guide whether we trust or want to collaborate with them (Erle et al., 2018). This suggests that even in the absence of objective cues, individuals may still follow discernible patterns in partner selection, driven by objectively irrelevant factors.

This gap in coalition theories stresses a significant limitation: they do not adequately account for how partner selection occurs in ambiguous environments. The present research examines how incidental cues, specifically through visuo-spatial perspective-taking (VSPT), influence coalition formation when static features are absent. The 2(1–1–1) game is an ideal scenario for studying these effects, as it removes economic factors and focuses on psychological influences on partner selection.

## Perspective-taking

When deciding whether to cooperate with someone, it is essential to understand whether our potential allies share intentions and motivations that align with our own. Individuals rely on cues, available information such as facial expressions, verbal statements, patterns of behavior, or contextual signals like resource distributions or strategic roles, to guide these perceptions. But how do individuals infer intentions or motivations from these cues? Perspective-taking plays a pivotal role in this process. Experts in the field have developed a consensual lexicon, defining perspective-taking as “the process by which one represents others’ mental states by adopting their perspective” (Quesque et al., 2024, p. 2). By adopting another’s perspective, individuals can discern their motives and respond appropriately in negotiations (Ku et al., 2015).

Perspective-taking is a form of mentalizing that encompasses cognitive, emotional, and perceptual states. All forms of perspective-taking rely on three core abilities: (a) recognizing that others have mental states, (b) understanding that these mental states may differ from one’s own, and (c) overcoming egocentrism to adopt a different literal (visuo-spatial) or metaphorical (psychological) point of view (Epley & Caruso, 2009; Erle & Topolinski, 2017; Quesque & Rossetti, 2020).

In practical terms, perspective-taking allows individuals to anticipate others’ moves in negotiations by considering how they might perceive the situation. For example, in a classic 5(4–3–2) game, the player holding 2 resources can put themselves in the perspective of the two other players and imagine what kinds of offers they would make. From this inference about the likely state of the other players, they would deduce that it will be more costly to form a coalition with the player holding 4 resources, and consequently approach the player holding 3 resources during the negotiation. Indeed, this is a common finding in the literature on coalition formation called the Strength-is-Weakness effect (Murnighan, 1991; Vinacke & Arkoff, 1957; Wissink et al., 2021, 2022). Previous research has shown that trait perspective-taking plays a role in this process and can improve coalition outcomes in such scenarios (Cantiani, van Beest, Cruijssen, et al., 2024; Cantiani et al., 2025), especially when the mental states of others are less predictable (Cantiani, van Beest, & Erle, 2024). Similarly, in different negotiation scenarios, perspective-taking enables individuals to make more strategic decisions by considering both their own goals and the priorities of the other party (Galinsky, Maddux et al., 2008).

However, all these studies deal with situations in which there are some cues that allow a perspective-taker to infer a mental state in the other players (e.g., their likely allocation preferences). But even in situations where there is no indication of the preferences and mental states of the other negotiation partners, we argue that perspective-taking can affect a negotiation because it has been proposed that perspective-taking plays a fundamental role in fostering social bonds, which can facilitate cooperation (Galinsky et al., 2005). Specifically, through perspective-taking, individuals experience a merging of self and other, seeing more of themselves in others while also adopting traits of the other person (Davis et al., 1996, 2004; Galinsky, Wang et al., 2008), which creates feelings of similarity, liking, and trust, which are essential for building cooperative relationships (Batson, Early et al., 1997; Batson, Polycarpou, et al., 1997; Erle et al., 2018). What is less understood are the mechanisms behind how perspective-taking creates these social effects, particularly when interacting with multiple social targets.

One hypothesis is that *embodiment*, the mental simulation of another’s perspective, drives these effects. This process, also called “perspective transformation” or “viewer rotation” (Kessler & Thomson, 2010; Kozhevnikov et al., 2006; Michelon & Zacks, 2006; Wraga et al., 2000; Zacks & Tversky, 2005), allows individuals to mentally rotate their body schema to adopt another’s viewpoint. This mechanism has been primarily demonstrated using VSPT tasks, where participants view a scene either from another person’s perspective (perspective-taking trials) or their own egocentric viewpoint (egocentric trials), and are asked to complete an action, such as grabbing an object. These tasks rely on level-2 VSPT, which requires imagining not just *what* another person sees (level-1 VSPT) but *how* they see it (Flavell et al., 1981). Evidence for embodiment in VSPT comes from performance declines when participants’ bodies are constrained or rotated incongruently with the target’s position (Kessler & Thomson, 2010; Surtees et al., 2013; Yu & Zacks, 2017). Virtual reality studies further support this, showing that even without visual feedback, vestibular sensations alone can hinder perspective-taking (Deroualle et al., 2015). Additionally, individuals often make mistakes based on their imagined perspective rather than their actual position, further supporting the idea that the body influences perspective-taking (Samuel et al., 2020).

Beyond blurring self-other boundaries and integrating motor representations (Samuel et al., 2020), studies have also shown that VSPT has social effects, as it increases feelings of similarity, liking, and trust toward the targets involved, often without participants being consciously aware of these effects (Erle & Topolinski, 2017; Erle et al., 2018). Embodiment, therefore, appears to be a crucial mechanism for explaining the social effects of perspective-taking. The merging of self and other perspectives through embodied simulation fosters social closeness, increasing liking, similarity, and trust. Of note, these factors can affect negotiation behaviors even in situations where access to the mental states of other players is difficult. Specifically, even in situations such as a 2(1–1–1) simple weighted majority game, where there is no way of discerning the intentions of the other two players based on static game parameters, the other players might plausibly differ in how much one likes them, finds them similar to the self, trustworthy, and so on. And level-2 VSPT might affect these perceptions via an embodied merging of the self and the other, and therefore also affect one’s willingness to form a coalition with specific players. However, currently, there is no empirical evidence supporting this second assumed pathway by which perspective-taking could affect coalition formation.

However, prior research has shown that not all individuals rely on embodied strategies. Some, known as “disembodiers,” use alternative methods that avoid embodiment (Samuel et al., 2023, 2024). These individuals solve VSPT tasks by reversing left-right mappings from their own perspective without embodying the other person’s viewpoint (Gardner et al., 2013) or by mentally rotating objects within the scene to match their egocentric orientation rather than adopting the other’s perspective (Wraga et al., 2000, 2003; Zacks & Tversky, 2005). Disembodiment is more frequently observed in men (Kaiser et al., 2008; Kessler & Wang, 2012; Kessler et al., 2014) as well as in individuals with social differences, such as those with schizotypy (Langdon & Coltheart, 2001) or autism (Conson et al., 2015; Hamilton et al., 2009; Pearson et al., 2016).

If embodiment is indeed the driving mechanism behind the social effects of perspective-taking, an important question arises: Do these effects manifest in disembodiers, and if so, how? Theoretically, disembodiers should not exhibit the same increases in perceived similarity, liking, or trust as those who use embodied perspective-taking. However, prior research has not empirically tested it, leaving a gap in understanding how perspective-taking strategies affect social outcomes.

## The present research

In the present experiments, participants completed two tasks consecutively: first, a VSPT task, followed by a 2(1–1–1) weighted majority coalition game. Crucially, we manipulated the VSPT task so that participants consistently engaged in perspective-taking with one of the two players, while remaining egocentric with the other. This setup allows perspective-taking to be induced through situational contingencies rather than explicit instructions, ensuring equal attention to all targets while only engaging in perspective-taking with some. Thus, it induces perceived similarity and likability for one other player, without giving away the crucial manipulation of the study.

Following the VSPT task, participants participated in a 2(1–1–1) coalition formation game with the two target individuals of the VSPT task. In a 2(1–1–1) game, there are no objective factors favoring one coalition partner over another. According to coalition theories, coalitions in this configuration should form equally among all possible pairs (AB = AC = BC). This symmetric setup allowed us to isolate psychological factors like perceived similarity from the economic factors central to coalition theories.

We hypothesize that, in the absence of clear objective cues for cooperation or competition, individuals will be more likely to form coalitions with partners whose perspectives they have taken, simply because they perceive these individuals as more likable and similar to the self. Contrary to economic theories of coalition formation, this would suggest that psychological factors also play a role in determining coalition behavior. Additionally, based on the idea that the social effects of perspective-taking are rooted in an embodied simulation of physical closeness (e.g., Erle & Topolinski, 2017), we predicted that perspective-taking’s impact on coalition partner selection would be evident only among individuals who engage in embodied perspective-taking. In contrast, disembodiers, who do not engage in this simulation during the VSPT task, should demonstrate no differences or would more frequently choose the egocentric target, as these trials are cognitively easier to solve or potentially due to self-bias effects (a tendency to favor stimuli related to oneself, Sui et al., 2012).

Thus, across five experiments, the present research seeks to make contributions in two areas. First, it advances coalition formation theory by investigating how perspective-taking influences partner selection when no objective cues distinguish potential partners—situations where major theories predict random selection. By identifying psychological mechanisms that drive partner preference in these cases, this research extends our understanding of coalition formation beyond purely economic factors to include social psychological influences. Second, it provides a more nuanced understanding of how perspective-taking fosters social closeness, a frequently replicated but poorly understood phenomenon. By examining whether strategies (embodied vs. disembodied) determine whether the social effects of VSPT emerge or not, we address what has been labeled a critical question in the fields of social cognition and perspective-taking (Quesque et al., 2024; Samuel et al., 2024).

## Open science practices

### Transparency

All de-identified data, analysis scripts, and materials are available at OSF (https://osf.io/ykj2x/). We preregistered hypotheses, planned analyses, and sample sizes for Experiments 2 (https://osf.io/mp29z/) and 3 (https://osf.io/7qesy/). An additional experiment (Experiment 2 *bis*) had a methodological error (lack of counterbalancing), so its results are not included, but the data are available on OSF (https://osf.io/2kf5u/).

We report all data, including any data exclusions, manipulations, and primary measures. The Supplemental Material provides a comprehensive list of all variables included in the experiments, along with the specific order in which they were measured. Additionally, it includes details on exploratory variables that were part of certain experiments but not addressed in the main manuscript. All relevant data are reported either in the manuscript or the Supplemental Material. All experiments were conducted with the approval of the Ethics Review Board at of the School of Social and Behavioral Sciences of Tilburg University.

### Power analyses

For Experiment 1, we conducted a post hoc sensitivity analysis using G*Power (Faul et al., 2007) to determine the minimum detectable effect size for the observed difference in coalition partner selection between two independent groups (*n* = 127 for the embodied strategy, *n* = 66 for the disembodied strategy). The analysis used a significance level (α) of .05 and aimed for 80% statistical power. The proportions for the two groups were set as *p1* = 0.58 (embodied strategy) and *p2* = 0.39 (disembodied strategy), reflecting the observed preference for the perspective-taking target. The analysis revealed that the minimum detectable difference in proportions (*p*1 − *p*2) was *OR* = 2.12. This value is below our observed effect size (*OR* = 2.15), indicating that the study was sufficiently powered to detect the difference.

For Experiment 2, we hypothesized that strategy would moderate coalition partner selection. To test this, we calculated the required sample size to achieve a power of (1 − β) = .80 in a logistic regression, using the partner selection ratio from Experiment 1. This resulted in a total of 250 participants (125 per strategy), determined using the R function rbinom. We preregistered that data collection would be terminated once *N* = 250 valid cases were obtained, or alternatively, when reaching a sample size of *N* = 500 participants. This approach was intended to balance the need for a sufficient number of valid cases with the avoidance of inefficiency and financial loss, given that we could not control factors such as the number of participants reporting each strategy during the VSPT task or the formation of grand coalitions. The second stopping rule was applied, and data collection continued until the sample size reached 500 participants.

In Experiment 3, we conducted a simulation-based power analysis using R to assess the moderating effect of strategy on coalition-forming intentions. Analyses were performed in R version 4.5.0 (R Core Team, 2024). Main packages included tidyverse (v2.0.0; Wickham et al., 2019), readxl (v1.4.3; Wickham & Bryan, 2025), writexl (v1.4.2; Ooms, 2023), dplyr (v1.1.4; Wickham et al., 2025), ggeffects (v1.5.0; Lüdecke, 2018), lme4 (v1.1-34; Bates et al., 2015), ggstatsplot (v0.12.0; Patil, 2021), lmerTest (v3.1-3; Kuznetsova et al., 2017), sjPlot (v2.8.15; Lüdecke, 2023), and effsize (v0.8.1; Torchiano, 2020). We averaged participant ratios from previous studies (Experiment 1, *M* = 2.93; Experiment 2, *M* = 2.71; Experiment 2 *bis*, *M* = 1.98), resulting in *M* = 2.54 embodiers to disembodiers. Using effect sizes from Erle et al. (2018) on trust (*d* = 0.34) and liking (*d* = 0.32), we simulated the embodied strategy condition. Since no data were available for the disembodied condition, we modeled two scenarios: one with no preference and one with the opposite effect. We estimated sample sizes of *N* = 190 for disembodiers and *N* = 483 for embodiers to achieve a power of (1 − β) = .80. The stopping rule for Experiment 3 was to collect data until 673 valid observations. However, due to the lack of control over the number of participants reporting each strategy, we estimated needing to recruit approximately *N* = 959 participants (*n* = 190 disembodiers, *n* = 473 embodiers, and *n* = 286 reporting both) based on previous ratios of 2.54 embodiers to 1 disembodier and 1.69 embodiers to 1 using both strategies. For Experiments 4a and 4b, where we hypothesized a main effect of VSPT on partner selection, a G*Power a priori analysis based on the meta-analytic effect sizes from the four previous experiments (where 55.6% preferred the perspective-taking target compared to the egocentric target, total *N* = 2,008) indicated that at least *N* = 498 participants were needed for 80% power.

### Exclusion criteria

We preregistered exclusion criteria for participants with low VSPT accuracy. A cumulative binomial test in R showed that achieving 4 or fewer correct trials (out of 16) was below chance (*p* < .039) in Experiments 1 to 3, while in Experiments 4a and 4b, the threshold was set at 10 correct trials (out of 32, *p* < .025).

For VSPT reaction time (RT) analysis, we excluded trials and participants with RTs outside the 1.5 IQR range (Tukey, 1977). This criterion was applied both at the trial level, removing slow and fast trials from the analysis for each participant, and at the subject level, excluding participants with unusually slow or fast reaction times from the sample. Only trials with correct responses were included in the RT analyses.

### Analytic strategy

Across all studies, we consistently followed the same analytic approach. To inspect whether participants completed the VSPT task as intended, we analyzed the discrepancy in reaction times and accuracy rates between target types (perspective-taking vs. egocentric) using “target type” as a fixed predictor of RTs/accuracy rates, respectively (formula: RTs/ACCs ~ target type + (target type | subject) + (1 | subject)). Due to non-convergence, only the random intercept remained in the final model, as specified in the preregistration.

To test our main hypotheses, that is, to assess the effect of perspective-taking on coalition partner selection and to explore whether strategies (embodied vs. disembodied) moderate this effect, we employed a logistic regression (formula: selected coalition partner ~ 1 + strategy) with “selected coalition partner” (1 = perspective-taking vs. 0 = egocentric target) as the binary dependent variable.

To test the effects of perspective-taking and strategy on money allocation (i.e., how much money individuals allocate to their selected coalition partner), we performed a linear regression analysis with opening offer as the continuous dependent variable, and the predictors “selected partner” (1 = perspective-taking vs. 0 = egocentric target), “strategy” (embodied vs. disembodied), as well as their interaction. We conducted a similar analysis on liking and similarity ratings. However, given that participants rated multiple targets, we conducted a linear mixed-effects model (formula: ratings ~ target type * strategy + (target type | subject) + (1 | subject)). Following Barr et al. (2013) guidelines, we also pre-registered to simplify the model structure in the event of non-convergence. Consequently, the final models retained only “subject” as the random effect (formula: ~1 | subject).

The predictor variable “strategy” was coded to distinguish between people who solved the VSPT task using an embodied strategy (“embodiers”) and those who did not (“disembodiers”). In Experiment 1, participants could indicate using either of these strategies or both. For the analysis, we excluded all participants who indicated using both strategies. From Experiment 2 onwards, we asked individuals who initially declared using both embodied and disembodied strategies to indicate their predominant approach, and this forced response was used to allocate them for the main analysis to either group of VSPT strategies.

## Experiment 1

Experiment 1 was designed to examine the influence of perspective-taking on coalition partner selection in a scenario where no objective cues to the other players’ intentions are present, and to determine whether this influence is moderated by the strategy (embodied vs. disembodied) used by participants when engaging in perspective-taking.

We hypothesized that individuals using an embodied strategy in perspective-taking would prefer to form coalitions with partners whose perspectives they adopted (perspective-taking target) rather than with those from whom they maintain an egocentric viewpoint (egocentric target). Conversely, we expected those employing a disembodied strategy in the VSPT task to show no preference, or a tendency to favor the egocentric targets. We anticipated that this pattern would also apply to liking and similarity ratings as well as money allocation.

### Method

#### Participants

*N* = 193 participants (*n* = 136 female, *n* = 54 males, *n* = 1 other, and *n* = 2 preferred not to say, *M*_age_ = 27.10, *SD*_age_ = 7.63), following the exclusion of *n* = 11 participants with accuracy rates significantly below the chance level, and removing *n* = 100 participants who reported using both cognitive strategies. Participants were recruited via Prolific Academic (Damer & Bradley, 2014). Out of this sample, 65.80% (*n* = 127) of participants reported using an embodied strategy when solving the VSPT task. The duration of the study was about 5 minutes, and participants were compensated with £0.50.

#### Procedure

After providing signed informed consent, participants were paired with two distinguishable pre-programmed partners (named Benson and Dunlop) that they were told they would be completing the remaining tasks of the experiment. They were informed that they would first complete 2 practice trials, followed by 16 experimental trials of a VSPT task, adapted from Kessler and Thomson (2010). Then they rated how much they liked and felt similar to each partner, and completed a coalition formation simulation (based on a 2(1–1–1) simple majority game; van Beest et al., 2003). After the main tasks, participants answered some questions about how they completed the VSPT task, provided basic demographic information (i.e., age and gender), were thanked, and debriefed.

#### Materials

##### VSPT task

The VSPT task was adapted from Kessler and Thomson (2010). In each trial, participants see a person seated behind a table at an angular disparity of 160° (clockwise or counterclockwise) from their position, with two objects placed on the table in front of them (i.e., a flower and a pistol). During every trial, participants were instructed to virtually “grab” one of these two objects from an instructed perspective using two response keys. The dependent variables of this task are participants’ mean RT and accuracy.

**Figure 1.**
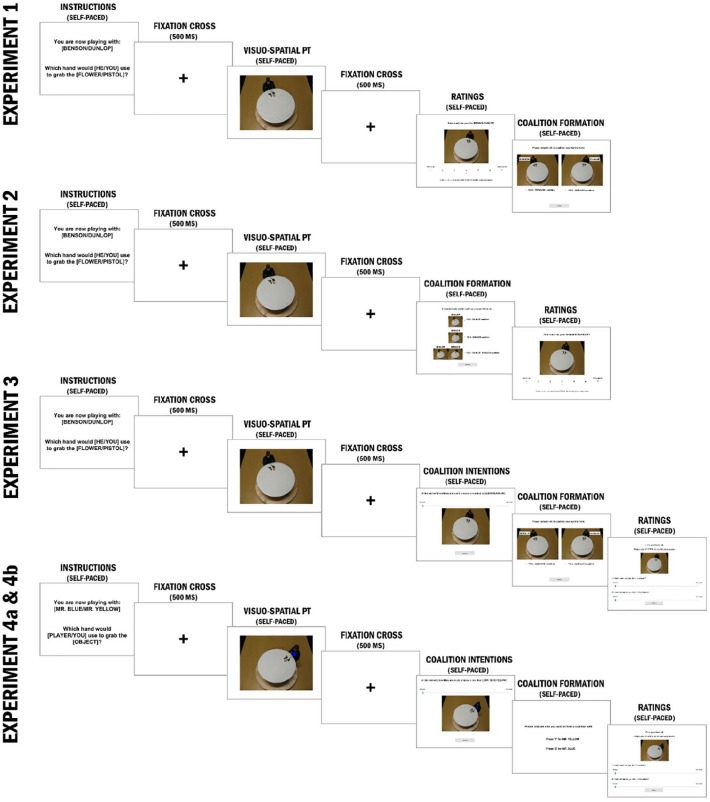
Experimental timelines of Experiments 1 to 4b and exemplary stimuli. *Note*. A full-size version of this figure is available at the following OSF link for a clearer view of the details: https://osf.io/ykj2x/.

As illustrated in Figure 1, at the beginning of every trial, participants received information about which of the two other players (Benson or Dunlop) they were playing with for this trial, which perspective they had to assume (the target person’s or their own egocentric perspective), and which object they had to grab. This instruction was displayed until participants pressed “A” or “L” to continue. Next, a fixation cross was presented for 500 ms, followed by an image of the target person at the table. Again, the screen was displayed until participants responded which hand would be used to grab the target object from the instructed perspective. Participants rendered their responses by pressing “A” when referring to the left hand, or “L” to the right hand (from either their or the target’s perspective). Whenever participants made a mistake, an error message was displayed for 2,500 ms.

Unbeknownst to participants, we created a contingency between the two target players and the perspective instructions to manipulate perspective-taking: participants consistently engaged in perspective-taking whenever one of the two other players was presented and remained consistently egocentric when the other was presented. To ensure the distinctiveness of the game partners, their angularity was kept constant throughout the experiment (e.g., Dunlop always appeared rotated clockwise, whilst Benson was rotated counterclockwise). Who the egocentric and the perspective-taking target was and at which angular disparity they were displayed, was counterbalanced across participants. The target objects were counterbalanced across trials.

To avoid potential confounds related to facial visibility, both the perspective-taking and egocentric targets were presented facing the participant, ensuring that both targets’ faces were equally visible throughout the task.

##### Liking and similarity ratings

After the VSPT task, participants indicated their liking (i.e., How much do you like [potential partner label]?) and perceived similarity (i.e., How similar do you feel to [potential partner label]?) of each of the game partners (i.e., Benson and Dunlop) on a scale ranging from 1 (*not at all*) to 7 (*very much*).

##### Coalition formation task

To create a situation without any economically relevant static features, we used a landowner paradigm developed by van Beest et al. (2003) to operationalize a 2(1–1–1) simple weighted majority game. During this game, the participant and the two game partners from the VSPT task (i.e., Benson and Dunlop) are taking the role of landowners who own a piece of land (i.e., 1 hectare each ). They are approached by a project developer who wants to buy 2 hectares for 25 million dollars. As none of the landowners possesses sufficient hectares to satisfy the developer’s demand, they need to form a coalition.

Participants were instructed to select one of the game partners and to propose an economic offer to that partner. Regarding the coalition partner selection, participants were given the possibility to form only small coalitions (i.e., to form a coalition with Benson or with Dunlop, but not with both). They then allocated the 25 million dollars between themselves and the selected coalition partner. After making their offer, participants were also required to specify their reservation price, which represented the minimum offer they would accept. The instructions stated, “*Please indicate the minimum amount from the 25 million budget that you would accept from your partner in order to agree to form a coalition*.”

##### Embodied vs. disembodied perspective-taking strategies

We asked participants to indicate which strategy they used to complete the VSPT task, as in previous research (Gardner et al., 2013; Gronholm et al., 2012). Participants who reported “*I imagined myself sitting in the partner’s position to judge what hand he would use from there*” were classified as *embodiers*. Conversely, participants who indicated, “*I imagined myself grabbing the object from my egocentric perspective and reversed the response I computed because the other person sat across the table*” were classified as *disembodiers*. Lastly, individuals who selected “*I used both strategies*” constituted a distinct category.

### Results

#### Perspective-taking performance

Prior research has robustly shown that individuals require more time and make more errors when engaging in VSPT compared to when they remain egocentric (Erle & Topolinski, 2015, 2017; Erle et al., 2018; Kessler & Rutherford, 2010; Kessler & Thomson, 2010; Surtees et al., 2013). Therefore, we expected to observe higher RTs and lower accuracy rates in perspective-taking trials relative to egocentric trials when participants carry out the VSPT task as intended.

##### Reaction times

The model’s total explanatory power was 42% (conditional *R*^2^ = .42), meaning that both the fixed effects and random effects together explain nearly half of the variance in reaction times. The variance explained by the fixed effects alone (marginal *R*^2^ = .06) was 6%. We found an effect of target type, β = 251.29, 95% CI [211.82, 290.75], *t*(2,049) = 12.49, *p* < .001. Consistent with prior studies, participants exhibited longer reaction times when adopting the viewpoint of the target (M = 1,107 ms, *SD* = 579 ms) compared to when maintaining an egocentric standpoint towards the target (*M* = 857 ms, *SD* = 519 ms).

##### Accuracy rates

The model’s total explanatory power was 21% (conditional *R*^2^ = .21), and the part related to the fixed effects alone (marginal *R*^2^ = .01) was 1%. As expected, accuracy rates were higher when maintaining an egocentric perspective towards the target (*M* = 0.74, *SD* = 0.24) than when taking the perspective of the target (*M* = 0.69, *SD* = 0.24), β = −.05, 95% CI [−0.08, −0.01], *t*(582) = −2.64, *p* = .009.

#### Coalition outcomes

##### Coalition partner selection

**Figure 2.**
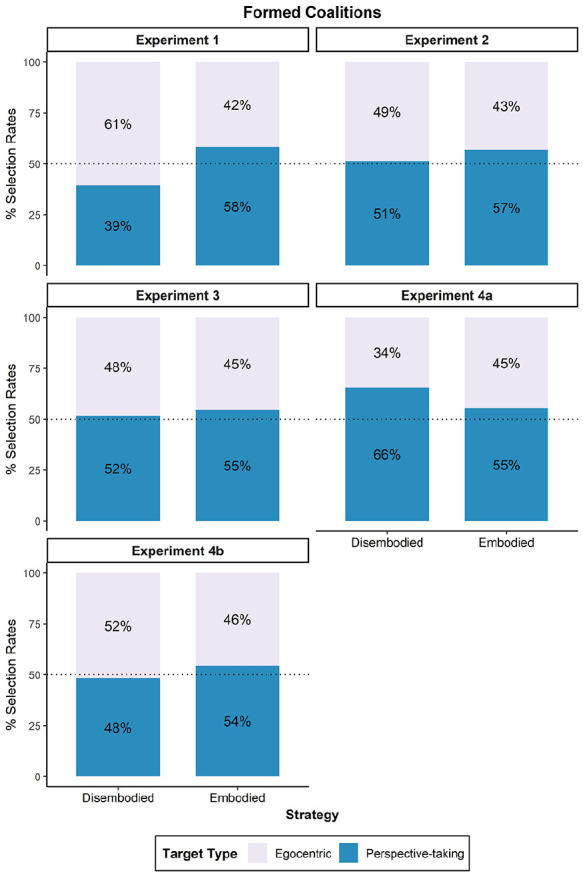
Partner selection rates across Experiments 1 to 4. *Note.* This graph compares the selection rates (*y*-axis) of the coalition partner whose perspective they adopted (perspective-taking target) to their selection of the coalition partner whose perspective was not adopted (egocentric target) based on the strategy (embodied vs. disembodied) used during the VSPT task. VSPT = visuo-spatial perspective-taking.

Overall, the model incorporating the strategy variable provided a significantly better explanation for the variation in partner selection compared to the model without it, χ^2^(1) = 6.23, *p* = .013, *R*^2^ = .02. The intercept was not significant, *OR* = 0.65, 95% CI [0.39, 1.06], *z* = −1.71, *p* = .087, indicating that there was no significant difference in the frequency of selection between the perspective-taking target (chosen by 51.8%, *n* = 100) and the egocentric target (chosen by 48.2%, *n* = 93). Moreover, we found an effect of strategy on coalition partner selection, *OR* = 2.15, 95% CI [1.18, 3.97], *z* = 2.47, *p* = .014. As shown in Figure 2, 58.3% of the individuals who reported using embodied perspective-taking decided to form a coalition with the perspective-taking target. In contrast, 60.6% of individuals employing a disembodied strategy selected the egocentric target. Despite these differences aligning with our hypotheses, post-hoc analyses conducted on each subgroup (embodiers vs. disembodiers) did not reach statistical significance when comparing selection rates against randomness, *OR*_Embodiers_ = 1.40, 95% CI [0.98, 1.99], *z* = 1.86, *p* = .064; *OR*_Disembodiers_ = 0.65, 95% CI [0.40, 1.06], *z* = −1.71, *p* = .087.

#### Perspective-taking outcomes

##### Liking and similarity ratings

**Figure 3.**
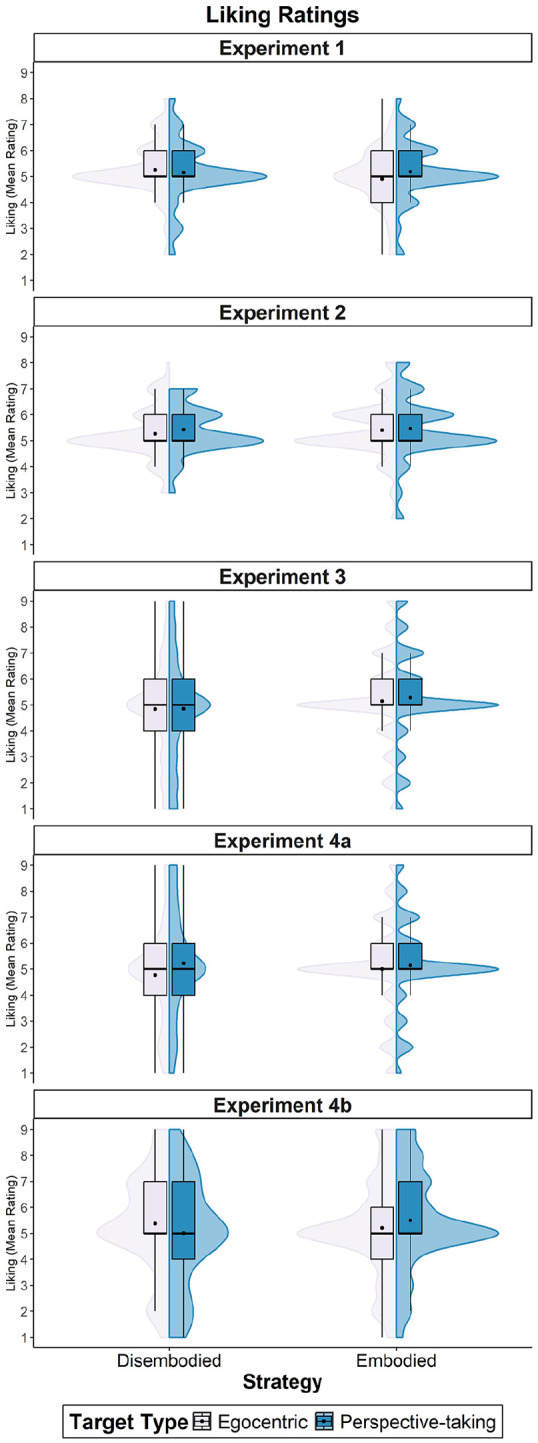
Mean liking ratings across Experiments 1 to 4. *Note.* This graph shows the mean liking ratings for the perspective-taking and egocentric targets, based on the strategy (embodied vs. disembodied) used during the VSPT task. VSPT = visuo-spatial perspective-taking.

For liking, the model’s total explanatory power was 68% (conditional *R*^2^ = .68), and the part related to the fixed effects alone (marginal *R*^2^ = .01) was 1%. There were no effects of target type, β = .11, 95% CI [−0.12, 0.33], *t*(380) = 0.94, *p* = .350, or strategy, β = −.04, 95% CI [−0.31, 0.38], *t*(380) = 0.21, *p* = .830. However, we found an interaction effect between target type and strategy, β = −.39, 95% CI [−0.66, −0.11], *t*(380) = −2.79, *p* = .006. As shown in Figure 3, embodiers reported higher levels of liking towards the partner whose perspective they had taken (*M* = 4.19; *SD* = 1.15), relative to the egocentric one (*M* = 3.91, *SD* = 1.14), β = .28, 95% CI [0.00, 0.57], *t*(252) = −1.97, *p* = .050. In contrast, disembodiers’ liking levels did not differ between the perspective-taking target (*M* = 4.15, *SD* = 1.18) and the egocentric target (*M* = 4.26, *SD* = 1.14), β = −.11, 95% CI [−0.51, 0.29], *t*(130) = 0.52, *p* = .601. Disembodiers liked the egocentric target more than embodiers did, β = .35, 95% CI [0.01, 0.69], *t*(191) = −2.04, *p* = .043, but there were no differences towards the perspective-taking target between embodiers and disembodiers, β = .04, 95% CI [−0.31, 0.39], *t*(191) = 0.21, *p* = .832.

**Figure 4.**
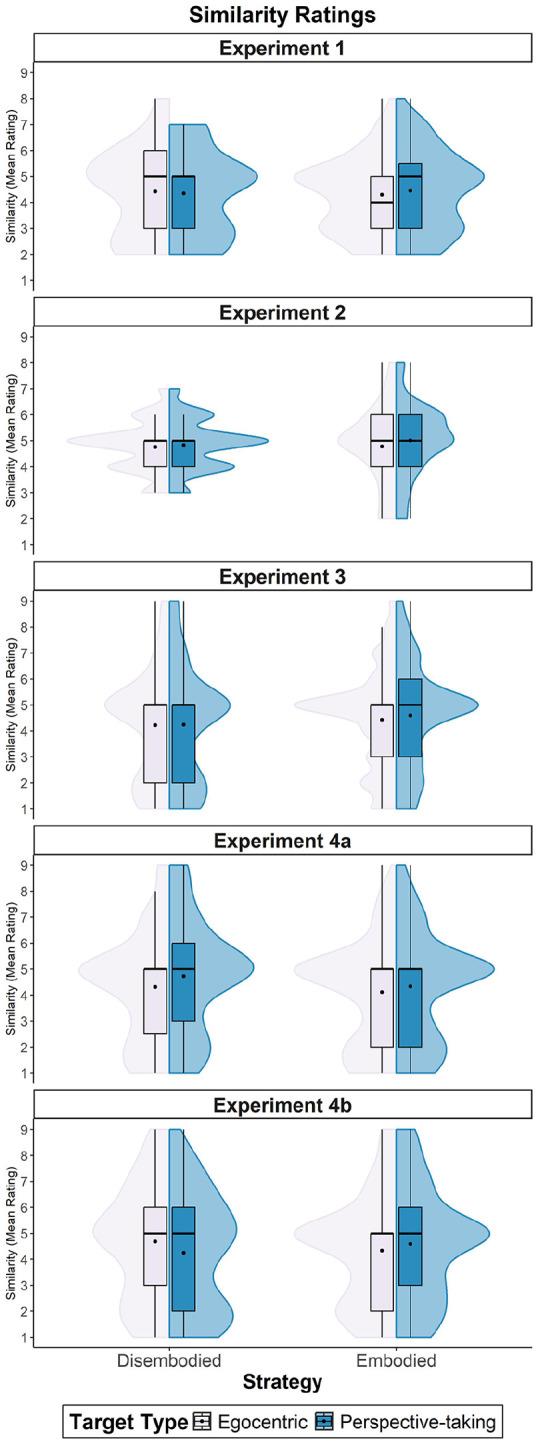
Mean similarity ratings across Experiments 1 to 4. *Note.* This graph shows the mean similarity ratings for the perspective-taking and egocentric targets, based on the strategy (embodied vs. disembodied) used during the VSPT task. VSPT = visuo-spatial perspective-taking.

As for similarity, the model’s total explanatory power was 76% (conditional *R*^2^ = .76), and the part related to the fixed effects alone (marginal *R*^2^ < .01) was <1%. Figure 4 shows that contrary to our hypothesis, none of the variables affected perceived similarity: β_Target type_ = .08, 95% CI [−0.19, 0.34], *t*(380) = 0.56, *p* = .573; β_Strategy_ = .10, 95% CI [−0.37, 0.57], *t*(380) = 0.42, *p* = .674, nor its interaction, β = −.23, 95% CI [−0.55, 0.10], *t*(380) = −1.36, *p* = .174.

### Discussion

The findings of Experiment 1 were partially consistent with our hypotheses, showing that perspective-taking influences coalition partner selection, and this effect is moderated by the strategies participants used. Specifically, embodied perspective-takers were more likely to choose partners whose perspectives they had considered, while disembodied participants seemed to favor the egocentric target. This preference likely stems from the reduced cognitive demands of the egocentric trials, as well as the influence of self-bias, where individuals tend to respond towards stimuli that are more closely related to themselves (Sui et al., 2012). This pattern was also reflected in liking ratings, with both groups showing greater liking towards the partners they selected.

However, no differences were found in perceived similarity or economic offers. This may be due to the reduced sample size after excluding 34% of participants who used both strategies, lowering the statistical power. To address this, we refined the strategy assessment in a follow-up study to include all participants. Additionally, we tested a potential boundary condition by allowing participants to form “grand coalitions” with both players, exploring whether the preference for perspective-taking targets in Experiment 1 was driven by the forced choice of a single partner.

## Experiment 2

The goals of Experiment 2 are twofold. First, we aimed to resolve ambiguities in the strategy assessment encountered in Experiment 1. To address this, we refined our strategy assessment by explicitly asking participants to indicate their predominant strategy, allowing us to clearly categorize each participant as using either an embodied or disembodied strategy during the VSPT task.

Second, we aimed to examine whether the effects observed in Experiment 1 persist when participants can form a grand coalition (i.e., a three-person coalition). This option introduces a more conservative test of preference strength, as participants can avoid choosing between two partners by selecting both. In Experiment 1, we showed that embodied perspective-taking led to more coalitions with the perspective-taking target when participants were forced to exclude one player. In this study, such an exclusion would be an active choice, indicating that an observed preference for the perspective-taking target would be less trivial.

### Method

#### Participants

Our sample consisted of *N* = 489 participants recruited via Prolific Academic (Damer & Bradley, 2014), following the exclusion of 18 participants with accuracy rates significantly below the chance level. To exclusively focus on the selection of the perspective-taking target over the egocentric target, we preregistered the exclusion of grand coalitions from the main analyses, such as partner selection, liking and similarity ratings, and money allocation. Unexpectedly, a large percentage of individuals (54.4%, *n* = 272) chose to form a grand coalition with both targets. As a result, we included only half of the initial sample (*N* = 223) in these analyses (*n* = 157 female, *n* = 61 male, and *n* = 5 other, *M*_age_ = 30.65, *SD*_age_ = 11.68). Of these, 153 participants were embodiers, representing 56.3% of the sample. The experiment duration was approximately 7 minutes, and participants received compensation of £0.75 for their participation.

#### Procedure

The procedure of Experiment 2 mirrored the procedure of Experiment 1, with only three key differences. First, the ratings (liking and similarity towards the targets) were presented after the coalition formation game to address the possibility that such ratings would influence coalition formation behavior. Second, participants had the option to select a grand coalition. In short, during the partner selection stage, participants had to indicate whether they preferred forming a coalition with only Dunlop, only Benson, or both of them. Third, the strategy assessment was refined. Consistent with our previous study, participants were first asked to specify the strategy they employed: embodied, disembodied, or both. However, for a more precise assessment, if a participant indicated using “both strategies,” they were further forced to indicate which of the two strategies was their predominant one. This categorization helped us classify them into one of the two main strategies we were investigating, either embodied or disembodied. Lastly, two more practice trials were included prior to the perspective-taking induction, for a total of four practice trials to enhance the VSPT performance, which had been lower in Experiment 1 compared to previously reported levels (e.g., Erle & Topolinski, 2017).

### Results

#### Perspective-taking performance

##### Reaction times

The model’s total explanatory power was 41% (conditional *R*^2^ = .41), and the part related to the fixed effects alone (marginal *R*^2^ = .05) was 5%. As expected, participants exhibited longer latency when adopting the target’s perspective (M = 1,109 ms, *SD* = 612 ms) than when maintaining an egocentric perspective towards the target (*M* = 852 ms, *SD* = 486 ms), β = 246.28, 95% CI [222.32, 270.23], *t*(5,246) = 20.15, *p* < .001.

##### Accuracy rates

The model’s total explanatory power was 35% (conditional *R*^2^ = .35), and the part related to the fixed effects alone (marginal *R*^2^ < .01) was <1%. Contrary to our expectations, participants’ accuracy levels did not differ between trials (Perspective-taking: *M* = 0.77, *SD* = 0.25; Egocentric: *M* = 0.76, *SD* = 0.23), β = .01, 95% CI [−0.02, 0.03], *t*(974) = 0.74 *p* = .457.

#### Coalition outcomes

##### Coalition partner selection

Overall, the model did not significantly enhance the prediction of partner selection, as evidenced by the negligible change in deviance from the null model, χ^2^(1) = .57, *p* = .449, *R*^2^ = .00. The intercept was not statistically significant, *OR* = 1.06, 95% CI [0.66, 1.70], *z* = 0.24, *p* = .811, suggesting that the perspective-taking target (chosen by 55.2%, *n* = 123) was not selected significantly more often than the egocentric target (chosen by 44.8%, *n* = 100). Moreover, strategy did not predict partner selection, *OR* = 1.24, 95% CI [0.70, 2.20], *z* = 0.76, *p* = .449. We observed that 56.9% (*n* = 87) of embodiers formed a coalition with the perspective-taking target, compared to 51.4% (*n* = 36) of disembodiers.

#### Perspective-taking outcomes

##### Liking and similarity ratings

When examining the effects of perspective-taking on both liking and similarity ratings, the model’s total explanatory power was 46% for liking (conditional *R*^2^ = .46) and of 51% for similarity (conditional *R*^2^ = .51) and the part related to the fixed effects alone (marginal *R*^2^ < .01) was of <1% for both variables. Neither the target type (Liking: β = −.17, 95% CI [−0.45, 0.10], *t*(440) = −1.22, *p* = .221, Similarity: β = −.07, 95% CI [−0.38, 0.24], *t*(440) = −0.45, *p* = .651), nor the strategy used (Liking: β = .04, 95% CI [−0.28, 0.36], *t*(440) = 0.26, *p* = .795, Similarity: β = .18, 95% CI [−0.20, 0.55], *t*(440) = 0.93, *p* = .353), nor the interaction between these variables (Liking: β = .11, 95% CI [−0.23, 0.44], *t*(440) = 0.63, *p* = .531, Similarity: β = −.16, 95% CI [−0.53, 0.22], *t*(440) = −0.83, *p* = .410) affected ratings.

### Discussion

In Experiment 2, we used a refined method for assessing VSPT strategies, which reduced the number of exclusions compared to Experiment 1. However, this improvement was offset by an unexpectedly high number of participants (54.4%) opting to form grand coalitions, which required their exclusion from our pre-registered analyses. Possibly due to this, we observed no effects of perspective-taking or strategies on partner selection and other outcome variables. This suggests that the influence of perspective-taking observed in Experiment 1 may not be as robust as we previously thought, at least when participants can form grand coalitions—an option that most participants preferred. This preference might suggest that when provided with a less restrictive decision-making environment, perspective-taking may lead to inclusivity (Cantiani, van Beest, Cruijssen, et al., 2024; Cantiani et al., 2025).

Interestingly, the behavior of disembodiers shifted between the two experiments. In Experiment 1, disembodiers showed a preference for the egocentric target, whereas in Experiment 2, their selections aligned more closely with embodiers, showing a slight, though non-significant, preference for the perspective-taking target. Given that post hoc analyses were non-significant in both experiments, it appears that disembodiers showed no clear statistical preferences in either Experiment 1 or Experiment 2.

On top of these concerns, while analyzing the conflicting results between Experiments 1 and 2, we identified a methodological issue that may have influenced partner selection: the position of the stimuli on the decision screen. As shown in Figure 1, at the partner selection stage in both experiments, participants were presented with images of two potential partners, one on the right and one on the left, and asked to select one for coalition formation. Regardless of whether the partner was the egocentric or perspective-taking target, participants tended to prefer the partner on the right side of the screen (see Supplemental Material for further details).

This laterality bias may be explained by the *body-specificity hypothesis* (Casasanto, 2009; Casasanto & Chrysikou, 2011), which suggests that individuals’ bodily characteristics affect their preferences and abstract thinking. For example, right-handed individuals tend to favor options on the right side, while left-handed individuals prefer those on the left—a pattern observed even in non-physical judgments (e.g., verbal decisions). Given that most of our participants were likely right-handed, this bias could have influenced their choices. However, it remains unclear whether the source of this bias originated during the VSPT trials, where participants repeatedly saw the target displayed on the right side, or during the decision stage due to the response format, when participants were presented with two identical avatars and had to select one.

At this point, we were left with conflicting findings: in Experiment 1, embodiers preferred the perspective-taking target, and disembodiers favored the egocentric target. In Experiment 2, however, neither of these effects was significant. It remains unclear whether these discrepancies were due to the option to form grand coalitions, disembodiers’ shifting behavior, the laterality bias, or potential issues with the manipulation itself. Although reaction times were consistent with our expectations, participants were not more accurate when adopting their own perspective compared to taking the target’s perspective. This suggests that the manipulation may not have effectively induced the intended differences in perspective-taking, thereby weakening its effects.

To address these issues, we decided to conduct a large-scale replication of Experiment 1, removing the grand coalition option and using the refined VSPT strategy classification from Experiment 2. This approach aimed to maximize the number of participants included in the analysis and provide a clearer understanding of typical disembodiers’ behavior. Additionally, by using a larger sample, we hoped to clarify whether the preference for the perspective-taking target is a general trend (indicating a main effect of perspective-taking on partner preferences) or whether it depends on the strategy used during the VSPT task (suggesting an interaction between perspective-taking and strategy).

## Experiment 3

This experiment closely replicated Experiment 1 with three main goals. First, to better understand the influence of perspective-taking on partner selection, we ensured a balanced representation of both embodiers and disembodiers. Second, to improve the effectiveness of the perspective-taking induction, we took steps to help participants better understand the different trial types. Third, to address the observed laterality bias in Experiment 1, we introduced a new measurement step before the partner selection stage, where participants rated their intentions to form a coalition with each target using a different response format. This was done to determine if the laterality bias (favoring the right side target) originated during the VSPT task and/or the partner selection stage. If the bias was due to the VSPT task, it would affect both intention ratings and partner selection. However, if it occurred only during the decision stage, the bias would appear solely in partner selection.

### Method

#### Participants

The final sample comprised *N* = 939 participants (*n* = 463 females, *n* = 459 males, *n* = 12 identified as other, and *n* = 5 preferring not to disclose; *M*_age_ = 38.79, *SD*_age_ = 13.62), following the exclusion of three participants with accuracy rates significantly below the chance level, adhering to our preregistered exclusion criteria. Initially, 48.1% (*n* = 452) reported utilizing an embodied strategy, 21.3% (*n* = 200) a disembodied strategy, and 30.6% (*n* = 287) both strategies. Participants who reported using both were then asked to indicate their predominant strategy, increasing the proportion of embodiers to 65.18% (*n* = 612) and disembodiers to 34.8% (*n* = 327). The study duration was approximately 9 min, and participants received a compensation of £1.15.

#### Procedure

The methodology closely replicated Experiment 1, except for the following modifications. To enhance the VSPT induction, we added four more practice trials before the perspective-taking induction, bringing the total to eight, and increased the overall number of trials to 32.

To address the laterality bias, we introduced a new measurement step before the partner selection stage, where participants answered the question: *“At this moment, how likely are you to propose a coalition to [*perspective-taking/egocentric target label*]?”* on a 9-point Likert scale, ranging from *not at all* to *very much*. This question was asked for each of the two potential partners, with the order counterbalanced across participants.

We excluded the reservation price question, as it did not yield informative results in the prior studies (these analyses are reported in the Supplemental Material).

### Results

#### Perspective-taking performance

##### Reaction times

The model’s total explanatory power was 45% (conditional *R*^2^ = .45) and the part related to the fixed effects alone (marginal *R*^2^ = .05) was 5%. As expected, participants exhibited significantly longer reaction times when engaging in perspective-taking (*M* = 961 ms, *SD* = 513 ms) compared to remaining egocentric towards a target (*M* = 743 ms, *SD* = 380 ms), β = 212.12, 95% CI [202.46, 221.79], *t*(20,535) = 43.00, *p* < .001.

##### Accuracy rates

The model’s total explanatory power was 60% (conditional *R*^2^ = .60), and the part related to the fixed effects alone (marginal *R*^2^ < .01) was <1%. As expected, participants made fewer errors when maintaining an egocentric perspective towards the target (*M* = 0.79, *SD* = 0.21) than when adopting the perspective of the target (*M* = 0.76, *SD* = 0.22), β = −.02, 95% CI [−0.04, −0.01], *t*(1,874) = −3.83, *p* < .001.

#### Coalition outcomes

##### Coalition partner intentions

In addition to our previous hypotheses, we also expected participants would show greater intentions to form coalitions with the perspective-taking target compared to the egocentric target. Additionally, we hypothesized that participants employing an embodied strategy during perspective-taking would exhibit greater intentions to coalesce with the perspective-taking target than with the egocentric target, and their intentions to coalesce with the perspective-taking target would be higher than those of disembodiers.

As pre-registered, we performed a linear mixed model analysis with restricted maximum likelihood estimation. The model included the fixed effects of target type (perspective-taking vs. egocentric) and strategy (embodied vs. disembodied), as well as their interaction, with random intercepts specified for the grouping variable subject. The model’s total explanatory power was 61% (conditional *R*^2^ = .61) and the part related to the fixed effects alone (marginal *R*^2^ = .01) was of 1%.

We found an effect of strategy on intentions to form a coalition, β = .42, 95% CI [0.15, 0.69], *t*(1,872) = 3.06, *p* = .002. Individuals employing an embodied strategy during the perspective-taking induction expressed stronger intentions to form coalitions (*M* = 4.55, *SD* = 1.95) than disembodiers (*M* = 4.07, *SD* = 2.06). Neither the target type, β = .02, 95% CI [−0.18, 0.21], *t*(1,872) = 0.15, *p* = .878, nor the interaction term, β = .10, 95% CI [−0.14, 0.34], *t*(1,872) = 0.79, *p* = .429, were predictive of intentions. Contrary to our expectations, embodiers did not significantly expressed greater intentions to form a coalition with the perspective-taking target (*M* = 4.61, *SD* = 1.91) relative to the egocentric target (*M* = 4.50, *SD* = 1.98).

To explore whether the laterality issue also emerged with this new measurement format, we extended the model by adding a new factor to control for target position—whether the target on the right side was the perspective-taking or egocentric target during the VSPT trials. The model’s total explanatory power was 61% (conditional *R*^2^ = .61), and the part related to the fixed effects alone (marginal *R*^2^ = .02) was 2%. Strategy remained the only predictor of intentions, β = .54, 95% CI [0.15, 0.93], *t*(1,872) = 2.74, *p* = .006, indicating that target position during the VSPT trials did not influence intention ratings.

##### Coalition partner selection

Overall, the model incorporating the strategy variable did not account significantly better for the variation in partner selection compared to the model without it, χ^2^(1) = .72, *p* = .397, *R*^2^ < .01. The intercept was not statistically significant, *OR* = 1.07, 95% CI [0.86, 1.33], *z* = 0.61, *p* = .543, meaning that, there were no significant differences in the frequency of formed coalitions with the perspective-taking target (53.6%, *n* = 503) relative to the egocentric target (46.4%, *n* = 436).

Furthermore, and contrary to our hypothesis, we did not find an effect of strategy on coalition partner selection, *OR* = 1.12, 95% CI [0.86, 1.47], *z* = 0.85, *p* = .397. As shown in Figure 2, 54.6% of the individuals who reported using embodied perspective-taking formed a coalition with the perspective-taking target. Similarly, 51.7% of individuals employing a disembodied strategy selected the perspective-taking target.

Lastly, we explored whether participants exhibited a preference for the partner presented on the right side during the selection stage by adding this factor to the model. Indeed, participants were about 52.3% less likely to select the perspective-taking target when the egocentric target is on the right side compared to when the perspective-taking target is on the right side, *OR* = 0.48, 95% CI [0.31, 0.74], *z* = −3.28, *p* = .001, indicating that the position of the targets influenced partner selection. Furthermore, the intercept emerged as significant, *OR* = 1.58, 95% CI [1.15, 2.20], *z* = 2.79, *p* = .005, showing a significant difference in the preference for the perspective-taking target over the egocentric one.

#### Perspective-taking outcomes

##### Liking and similarity ratings

The model’s total explanatory power was 68% for liking (conditional *R*^2^ = .68) and 73% for similarity (conditional *R*^2^ = .73), and the part related to the fixed effects alone (marginal *R*^2^ = .01) was 1% for both variables. In line with the pattern observed for intentions to form a coalition, we found that only the strategy used during the VSPT task predicted both liking and similarity rates towards the potential coalition partners (Liking: β = .49, 95% CI [0.25, 0.73], *t*(1,872) = 3.97, *p* < .001; Similarity: β = .35, 95% CI [0.08, 0.62], *t*(1,872) = 2.56, *p* = .011). Overall, embodiers provided higher rates than disembodiers (Liking: *M*_Embodiers_ = 4.21, *SD*_Embodiers_ = 1.73; *M*_Disembodiers_ = 3.84, *SD*_Disembodiers_ = 1.92; Similarity: *M*_Embodiers_ = 3.51, *SD*_Embodiers_ = 1.95; *M*_Disembodiers_ = 3.22, *SD*_Disembodiers_ = 2.17).

### Discussion

In Experiment 3, we aimed to address three main goals: improve the effectiveness of the perspective-taking manipulation, address the issue of laterality bias, and further clarify the influence of perspective-taking strategies on partner selection.

Concerning the laterality bias, a consistent preference for the partner presented on the right side during the partner selection stage was observed once again. However, this bias was not found in the intention ratings, which used a different response format. This suggests that the laterality bias is likely driven by the dichotomous choice format used during partner selection, rather than the target’s position during the VSPT task itself. If the bias had stemmed from the VSPT task, we would have expected it to influence both the selection stage and the intention ratings.

Regarding the influence of perspective-taking on partner selection, contrary to our hypothesis, we did not observe a significant interaction effect between strategy and partner selection. While there was a trend favoring the selection of the perspective-taking target as a partner, this difference was not statistically significant. Instead, we identified an effect of strategy across nearly all key outcomes—intentions to form a coalition, liking, and perceived similarity. Individuals employing an embodied strategy reported increased intentions, liking, and perceived similarity towards both targets, not exclusively towards the perspective-taking target as initially hypothesized.

This deviation from our expectations raised concerns about the effectiveness of our manipulation. While the manipulation did yield the expected differences in reaction times and accuracy, the effect sizes were smaller than previously reported, possibly insufficient to influence partner selection behavior. At this point, we were uncertain whether the measurement tools were sensitive enough to accurately capture these dimensions, or whether the perspective-taking strategy itself had less of an influence on partner selection than we originally anticipated.

Consequently, we redesigned subsequent studies to enhance the perspective-taking induction, ensuring it robustly influences the outcomes of interest. Additionally, we incorporated an alternative response format for partner selection to eliminate the laterality bias (Casasanto, 2009).

## Experiment 4

Given the issues in Experiment 3, Experiments 4a and 4b introduced both methodological and hypothesis changes. In terms of design, we adjusted the perspective-taking manipulation to increase the distinction between target trials, which may have contributed to the null effects. Additionally, the response format was fully replaced to eliminate the laterality bias that persisted in partner selection.

### Experiment 4a

As Experiment 3 still showed laterality bias in how people chose coalition partners, Experiment 4a implemented three main adjustments to reduce any arbitrary preferences participants might have for the two targets. First, during the perspective-taking induction, the targets were presented in various locations to avoid lateral biases that might influence participants’ decisions. Second, we replaced the response format for partner selection, introducing a new format that eliminated lateral cues entirely. Finally, to further reduce potential preference-related biases, we assigned a unique color to each target, providing a more neutral way to distinguish between them than names.

#### Method

##### Participants

The final sample comprised *N* = 489 participants (*n* = 233 females, *n* = 244 males, *n* = 12 identified as other; *M*_age_ = 40.08, *SD*_age_ = 14.16), following the exclusion of three participants with low accuracy rates significantly below the chance level, seven participants who reported being colorblind, and one participant due to missing data on relevant variables. Among these participants, 69.1% (*n* = 338) reported using an embodied strategy. The study lasted around 10 minutes, and participants received a compensation of £1.25.

##### Procedure

This experiment followed the procedure of Experiment 3, incorporating three main changes.

First, we assigned specific colors to each target for easier identification. Half of the participants were introduced to the perspective-taking target as Mr. Blue and the egocentric target as Mr. Yellow (with colors counterbalanced across participants). Each target’s t-shirt displayed a circle of their corresponding color (e.g., Mr. Yellow had a yellow circle on his t-shirt).

Second, to eliminate left-right bias in partner selection, we replaced the horizontal presentation of targets with a vertical text format (see Figure 1). Participants selected their coalition partner by pressing specific keys: “Y” for Mr. Yellow or “B” for Mr. Blue. This change was implemented based on Casasanto’s *body-specificity hypothesis* (2009), which suggests that individuals associate positive concepts with their dominant side. By presenting options vertically, we avoided any inherent left-right preference that could influence partner selection.

Third, during the VSPT task, we expanded the possible positions where targets could appear. Casasanto (2009) findings suggest that perceived “leftness” or “rightness” influences decision-making. To avoid either target being consistently perceived as left or right, we added more positions so that both targets now appeared at various angles (120°, 160°, 220°, and 240°) instead of being fixed on the left (160°) or right (200°). This modification aimed to prevent participants from associating either target with a specific side or position, further reducing potential laterality effects.

#### Results

##### Perspective-taking performance

###### Reaction times

The model’s total explanatory power was 49% (conditional *R*^2^ = .49), and the part related to the fixed effects alone (marginal *R*^2^ = .03) was 3%. As expected, participants exhibited significantly longer reaction times when engaging in perspective-taking (M = 1,194 ms, *SD* = 630 ms) compared to remaining egocentric towards a target (*M* = 954 ms, *SD* = 529 ms), β = 221.90, 95% CI [204.75, 239.05], *t*(10,206) = 25.36, *p* < .001.

###### Accuracy rates

The model’s total explanatory power was 50% (conditional *R*^2^ = .50), and the part related to the fixed effects alone (marginal *R*^2^ < .01) was <1%. Contrary to our expectations, participants’ accuracy rates did not differ significantly between trials (Perspective-taking: *M* = 0.73, *SD* = 0.24; Egocentric: *M* = 0.72, *SD* = 0.24), β = .01, 95% CI [−0.01, 0.03], *t*(974) = 0.85, *p* = .398).

##### Coalition outcomes

###### Coalition partner intentions

The model’s total explanatory power was 41% (conditional *R*^2^ = .41), and the part related to the fixed effects alone (marginal *R*^2^ < .01) was <1%. In contrast to our hypothesis, none of the predictors showed a significant effect on intentions to form a coalition, β_Target Type_ = .26, 95% CI [−0.08, 0.61], *t*(972) = 1.52, *p* = .130; β_Strategy_ = .16, 95% CI [−0.22, 0.53], *t*(972) = 0.81, *p* = .419; β_Target × Strategy_ = −.04, 95% CI [−0.46, 0.37], *t*(972) = −0.20, *p* = .838. Embodiers did not express significantly greater intentions to form a coalition with the perspective-taking target (*M* = 4.36, *SD* = 1.86) relative to the egocentric target (*M* = 4.14, *SD* = 1.91). Embodiers also did not express greater intentions to form coalitions with the perspective-taking target than disembodiers did (*M* = 4.25, *M* = 2.11).

###### Coalition partner selection

The model incorporating the strategy variable provided a significantly better explanation for the variation in partner selection compared to the model without it (χ^2^(1) = 4.56, *p* = .033, *R*^2^ = .01). The intercept was significant (*OR*_Intercept_ = 1.90, 95% CI [1.37, 2.68], *z* = 3.76, *p* < .001). Meaning that more coalitions were formed with the perspective-taking target (58.5%, *n* = 286) relative to the egocentric target (41.5%, *n* = 203). Furthermore, we found an effect of strategy, *OR* = 0.65, 95% CI [0.43, 0.97], *z* = −2.12, *p* = .034. Surprisingly, as depicted in Figure 2, 65.5% of the disembodiers formed a coalition with the perspective-taking target, whereas 55.6% of embodiers selected this target. Post-hoc analyses showed that the preference for the perspective-taking target over the egocentric target only reached statistical significance among disembodiers, *OR*_Disembodiers_ = 1.54, 95% CI [1.01, 2.36], *z* = 2.00, *p* = .046; *OR*_Embodiers_ = 1.19, 95% CI [0.93, 1.52], *z* = 1.37, *p* = .171.

##### Perspective-taking outcomes

###### Liking and similarity ratings

For liking, the model’s total explanatory power was 41% (conditional *R*^2^ = .41), and the part related to the fixed effects alone (marginal *R*^2^ < .01) was <1%. We found that strategy alone did not impact the ratings, β = −.08, 95% CI [−0.43, 0.27], *t*(972) = −0.45, *p* = .652. Instead, only target type predicted liking ratings, β = −.45, 95% CI [−0.77, −0.13], *t*(972) = −2.80, *p* = .005. Individuals reported feeling more liking towards the perspective-taking target (*M* = 4.17, *SD* = 1.79) compared to the egocentric target (*M* = 3.93, *SD* = 1.84). Contrary to our predictions, this effect remained consistent regardless of the strategy employed, as the interaction was not significant, β = .31, 95% CI [−0.07, 0.69], *t*(972) = 1.58, *p* = .115.

Concerning similarity rates, the model’s total explanatory power was 55% (conditional *R*^2^ = .55), and the part related to the fixed effects alone (marginal *R*^2^ < .01) was of <1%. Individuals perceived themselves as more akin to the perspective-taking target (*M* = 3.46, *SD* = 2.13) in comparison to the egocentric target (*M* = 3.18, *SD* = 2.05), as indicated by an effect of target type, β = −.41, 95% CI [−0.73, −0.09], *t*(972) = −2.53, *p* = .012. However, this effect was not modulated by the strategy, β = −.39, 95% CI [−0.80, 0.01], *t*(972) = −1.93, *p* = .054, nor were the ratings affected by the strategy alone, β = .19, 95% CI [−0.19, 0.57], *t*(972) = 0.97, *p* = .334.

#### Discussion

Experiment 4a suggested that the perspective-taking induction did not work as expected, as accuracy rates remained low despite the improvements made to the method to enhance target identification. To address this, we conducted a follow-up experiment (Experiment 4b) to correct these issues.

### Experiment 4b

This study replicated Experiment 4a with one key modification: instead of a trial-based perspective-taking induction, we utilized a block design methodology. This change was intended to lessen the cognitive load associated with switching tasks and to enhance the expected effects associated with each perspective.

#### Method

##### Participants

The sample comprised *N* = 499 participants (*n* = 246 females, *n* = 246 males, *n* = 6 identified as other, and *n* = 1 preferring not to disclose; *M*_age_ = 38.75, *SD*_age_ = 13.37), following the exclusion of four participants with low accuracy rates, and three colorblind participants. Within this sample, 71.3% (*n* = 356) of participants reported employing an embodied strategy. The study had a duration of approximately 10 minutes, and participants were compensated with £1.25.

##### Procedure

This experiment is an exact replication of Experiment 4a, differing only in its experimental design. Unlike the trial-based approach used previously, this version employs a block design methodology. This change follows previous studies that have indicated that completing perspective-taking versus egocentric trials in separate blocks provides more cognitive control, as it allows participants to deliberately focus on a single perspective throughout the block (e.g., Surtees et al., 2016). These studies have shown that reaction times are shorter, suggesting that participants can resolve tasks more easily, and at least the accuracy rates in egocentric trials are better. For this reason, we reasoned that the mixed blocks used in previous experiments, which involve constantly changing perspectives, not only increase switching costs but may also diminish the well-established advantage of the egocentric standpoint. Consequently, grouping trials corresponding to specific conditions, in this case, perspectives, could potentially reduce the cognitive demand of switching and amplify the expected effects associated with each perspective. The order of the blocks was counterbalanced between participants.

#### Results

##### Perspective-taking performance

###### Reaction times

The model’s total explanatory power was 41% (conditional *R*^2^ = .21), and the part related to the fixed effects alone (marginal *R*^2^ = .04) was 4%. As expected, participants exhibited significantly longer reaction times when engaging in perspective-taking (M = 1,084 ms, *SD* = 596 ms) compared to remaining egocentric towards a target (*M* = 845 ms, *SD* = 456 ms), β = 231.18, 95% CI [214.86, 247.50], *t*(10,607) = 27.77, *p* < .001.

###### Accuracy rates

The model’s total explanatory power was 54% (conditional *R*^2^ = .54), and the part related to the fixed effects alone (marginal *R*^2^ < .01) was <1%. Contrary to our expectations, accuracy rates were significantly higher for individuals adopting the perspective of the target (*M* = 0.77, *SD* = 0.20) than when maintaining an egocentric perspective towards the target (*M* = 0.75, *SD* = 0.23), β = .02, 95% CI [0.00, 0.04], *t*(994) = 2.16, *p* = .031.

##### Coalition outcomes

###### Coalition partner intentions

The model’s total explanatory power was 45% (conditional *R*^2^ = .45), and the part related to the fixed effects alone (marginal *R*^2^ < .01) was <1%. None of the predictors showed a significant effect on intentions to form a coalition, β_Target type_ = −.06, 95% CI [−0.40, 0.29], *t*(992) = −0.31, *p* = .753; β_Strategy_ = 0.05, 95% CI [−0.34, 0.45], *t*(992) = 0.27, *p* = .787, nor its interaction, β = .38, 95% CI [−0.03, 0.79], *t*(992) = 1.80, *p* = .072.

However, planned post-hoc comparisons showed that embodiers expressed greater intentions to form a coalition with the perspective-taking target (*M* = 4.60, *SD* = 1.98) relative to the egocentric target (*M* = 4.27, *SD* = 1.99), β = .32, 95% CI [0.11, 0.54], *t*(708) = 2.94, *p* = .003. And their intentions to form coalitions with the perspective-taking target were higher than those of disembodiers (*M* = 4.17, *SD* = 2.14), β = .43, 95% CI [0.03, 0.82], *t*(497) = 2.13, *p* = .033.

###### Coalition partner selection

Overall, the model incorporating the strategy variable did not account better for the variation in partner selection compared to the model without it (χ^2^(1) = 1.59, *p* = .207, *R*^2^ < .01). The intercept was not significant, *OR* = 0.93, 95% CI [0.67,1.29], *z* = −0.42, *p* = .676, indicating no significant differences in the frequency of formed coalitions with the perspective-taking target (52.7%, *n* = 263) relative to the egocentric target (47.3%, *n* = 236). Furthermore, we did not find an effect of strategy on coalition partner selection, *OR* = 1.28, 95% CI [0.87, 1.90], *z* = 1.26, *p* = .207. As shown in Figure 2, 54.5% of the individuals who reported using embodied perspective-taking formed a coalition with the perspective-taking target. Whereas 48.2% of individuals employing a disembodied strategy selected the same target.

##### Perspective-taking outcomes

###### Liking and similarity ratings

The model’s total explanatory power was 46% for liking (conditional *R*^2^ = .46) and of 57% for similarity (conditional *R*^2^ = .57), and the part related to the fixed effects alone (marginal *R*^2^ < .01) was of <1% for both variables. We found effects of target type on both liking, β = .37, 95% CI [0.03, 0.71], *t*(992) = 2.16, *p* = .031, and similarity ratings, β = .42, 95% CI [0.09, 0.75], *t*(992) = 2.48, *p* = .013. The effect of strategy was significant for liking rates, β = .49, 95% CI [0.11, 0.87], *t*(992) = 2.54, *p* = .011, but not for similarity reports, β = .34, 95% CI [−0.08, 0.77], *t*(992) = 1.59, *p* = .113.

Moreover, there were interaction effects between target type and strategy on both liking, β = −.66, 95% CI [−1.06, −0.26], *t*(992) = −3.25, *p* = .001, and perceived similarity, β = −.67, 95% CI [−1.07, −0.28], *t*(992) = −3.36, *p* < .001. As shown in Figures 3 and 4, embodiers reported higher levels of liking and similarity towards the perspective-taking target (Liking: β = .29, 95% CI [0.07, 0.50], *t*(708) = 2.65, *p* = .008; Similarity: β = .25, 95% CI [0.05, 0.46], *t*(708) = 2.39, *p* = .017), while disembodiers reported greater liking and similarity towards the egocentric target (Liking: β = .37, 95% CI [0.04, 0.70], *t*(282) = 2.19, *p* = .029; Similarity: β = .42, 95% CI [0.08, 0.76], *t*(282) = 2.41, *p* = .017). The perspective-taking target received higher sympathy ratings from embodiers compared to disembodiers, β = .49, 95% CI [0.11, 0.87], *t*(495) = 2.53, *p* = .012, but there was no difference in similarity ratings, β = .34, 95% CI [−0.09, 0.77], *t*(495) = 1.56, *p* = .120. For the egocentric target, sympathy and similarity ratings did not differ between groups (Liking: β = −.17, 95% CI [−0.55, 0.22], *t*(495) = −0.84, *p* = .399; Similarity: β = −.34, 95% CI [−0.76, 0.08], *t*(497) = −1.60, *p* = .111). task.

#### Discussion

In Experiments 4a and 4b, we sought to refine the perspective-taking manipulation and reduce potential laterality effects on partner selection. Although we successfully addressed the laterality bias by removing all lateral cues from the experiments, performance during the manipulation remained problematic. In Experiment 4a, there were no significant differences in accuracy rates between the egocentric and VSPT trials, and in Experiment 4b, the results were opposite to our expectations, leading us to question whether our manipulation was fully effective.

Regarding our hypotheses, some findings did align with our predictions. In Experiment 4b, participants embodying the perspective-taking target showed stronger intentions to form coalitions, but these intentions did not translate into actual partner selection. Moreover, the proposed mechanisms of liking and perceived similarity produced mixed outcomes across the experiments.

Across the series of five experiments, we encountered various challenges, including issues with the research design and sample sizes. While the design issues were eventually resolved, participant exclusions led to smaller sample sizes in some studies, with some studies having around 200 participants while others had up to 900. Additionally, the direction of effects varied: embodiers preferred the perspective-taking target, sometimes showed no preference, or, in one instance, favored the egocentric target. These inconsistencies, along with the use of a homogenous setup across all experiments, led us to conduct a meta-analysis to gain a clearer understanding of the overall patterns and to explore potential moderating factors within the data.

## Meta-analytical effects

As our series of experiments progressed, it became increasingly clear that neither the initial pattern we pursued (an interaction between perspective-taking and strategy, see Table 1 for an overview), nor the pattern that we discovered most often along the way (a perspective-taking main effect) was conclusively supported by all studies. Given these inconsistencies and the unresolved accuracy issues, it remains difficult to draw firm conclusions from the individual studies.

**Table 1.** Summary of results across experiments and meta-analysis.

Experiment	Coalition outcomes	Perspective-taking outcomes		Perspective-taking performance	
	Coalition partner selection	Perceived liking	Perceived similarity	Reaction times	Accuracy rates
Experiment 1	/✓	✓	✗	✓	✓
Experiment 2	✗	✗	✗	✓	✗
Experiment 3	✗	✗	✗	✓	✓
Experiment 4a	/✓	/✓	/✓	✓	✗
Experiment 4b	✗	✓	✓	✓	✗
Meta-Analysis	/✓	✓	✓	✓	✓

*Note*. ✓ = evidence consistent with the hypothesis; ✗ = no support for the hypothesis; /✓ = evidence consistent with preference for perspective-taking over egocentric target, but not modulated by the strategy.

As a result, we decided to conduct a one-stage Individual Patient Data (IPD) meta-analysis (Kontopantelis, 2018) to synthesize the data and better guide our interpretation. This approach allowed us to identify consistent patterns and clarify the effect sizes on each of the dependent variables more accurately.

We included all data from the five experiments (Experiments 2–4) with refined strategy assessments. For Experiment 1, we only incorporated data from participants who reported using either an embodied or a disembodied strategy, excluding those who indicated the use of both strategies. The model included target type and strategy, along with their interaction, as fixed effects. Moreover, the model incorporated a nested structure for subjects within experiments [(1 | Exp/subject)] as random effects. This structure accounts for shared variance within experiments that is not explained by the fixed effects, as subjects might behave more similarly within the same experiment.

### Perspective-taking performance

#### Reaction times

The one-stage IPD meta-analysis to examine the effects of perspective-taking on reaction times included *N* = 45,822 observations, from *N* = 2,236 subjects, and *K* = 5 studies. Overall, this model accounted for 46% of the total variance (Pseudo-*R*^2^ = .46), with level-1 ICC_subjects_ = 0.42, and level-2 ICC_experiments_ = 0.02, indicating that 42% of variance is attributable to differences between subjects within experiments and only 2% to differences between the experiments themselves.

**Figure 5.**
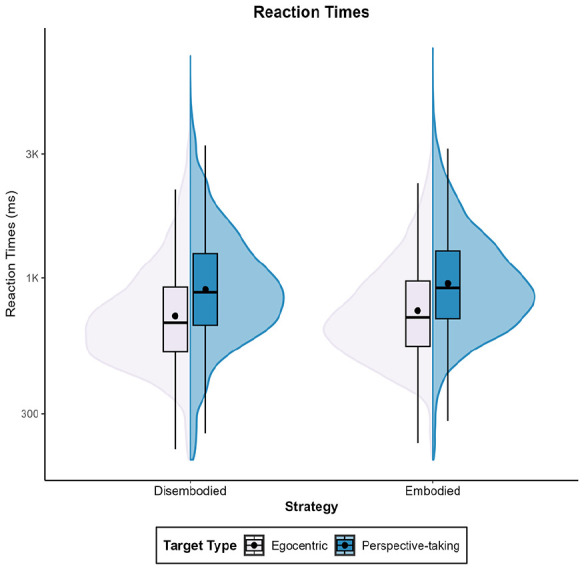
Mean reaction times across Experiments 1 to 4. *Note.* The *y*-axis is on an exponential scale. This graph visualizes the mean reaction times for the perspective-taking trials (when engaging in perspective-taking) and the egocentric trials (when assuming an egocentric viewpoint), as a function of the strategy (embodied vs. disembodied) used during the VSPT task. VSPT = visuo-spatial perspective-taking.

As shown in Figure 5, the results revealed an effect for target type, β = 225.21, 95% CI [212.00, 238.41], *t*(45,815) = 33.42, *p* < .001. Participants took significantly longer to respond when perspective-taking (M = 1,057 ms, *SD* = 578) compared to maintaining an egocentric viewpoint (*M* = 821 ms, *SD* = 448 ms). Moreover, there was an effect of strategy, β = 35.83, 95% CI [3.63, 68.04], *t*(45,815) = 2.18, *p* = .029, where embodiers took longer (*M* = 949 ms, *SD* = 525 ms) compared to disembodiers (*M* = 915 ms, *SD* = 542 ms). Lastly, the interaction between target type and strategy was not significant, β = −.56, 95% CI [−16.43, 15.32], *t*(45,815) = −0.07, *p* = .945, suggesting that the effect of target type on reaction was not moderated by strategy.

#### Accuracy rates

The one-stage IPD meta-analysis to examine the effects of perspective-taking on accuracy rates included *N* = 4,686 observations, from *N* = 2,340 subjects, and *K* = 5 studies. Overall, this model accounted for 52% of the total variance (Pseudo-*R*^2^ = .52). The intraclass correlation coefficients (ICCs) showed that individual differences (level-1 ICC_subjects_ = 0.50) account for half of the variance, while differences between experiments (level-2 ICC_experiments_ = 0.01) contribute minimally.

**Figure 6.**
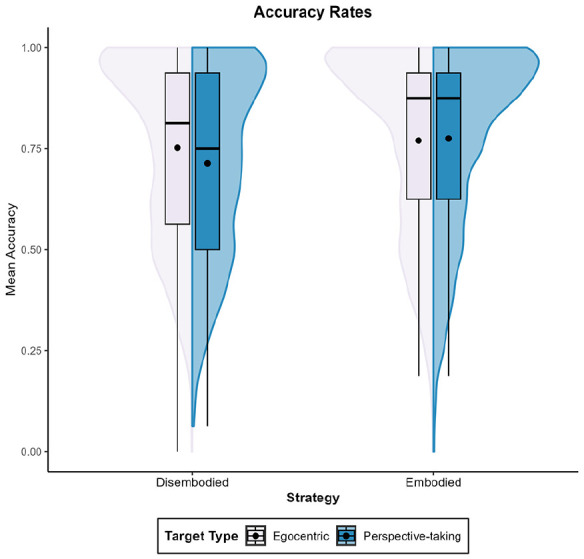
Mean accuracy rates across Experiments 1 to 4. *Note.* This graph visualizes the mean accuracy rates for the perspective-taking trials (when engaging in perspective-taking) and the egocentric trials (when assuming an egocentric viewpoint), as a function of the strategy (embodied vs. disembodied) used during the VSPT task. VSPT = visuo-spatial perspective-taking.

The results revealed an effect of target type, β = −.04, 95% CI [−0.06, −0.02], *t*(4,679) = −4.85, *p* < .001, while the effect of strategy was not significant, β = .02, 95% CI [−0.01, 0.04], *t*(4,679) = 1.89, *p* = .058. Importantly, there was an interaction between target type and strategy, β = .04, 95% CI [0.02, 0.06], *t*(4,679) = 4.51, *p* < .001. Results of post-hoc tests, detailed in Figure 6, showed that disembodiers were more accurate in egocentric trials (*M* = 0.75, *SD* = 0.23) than in perspective-taking trials (*M* = 0.71, *SD* = 0.23), β = −.04, 95% CI [−0.05, −0.02], *t*(1,509) = −4.85, *p* < .001. However, embodiers did not differ between trials (Perspective-taking: *M* = 0.78, *SD* = 0.22, Egocentric: *M* = 0.77, *SD* = 0.23), β = .01, 95% CI [−0.01, 0.02], *t*(3,167) = 0.91, *p* = .361. Furthermore, embodiers showed higher accuracy than disembodiers in both perspective-taking, β = .06, 95% CI [0.04, 0.08], *t*(2,338) = 6.31, *p* < .001, and egocentric trials, β = .02, 95% CI [0.00, 0.04], *t*(2,338) = 2.06, *p* = .040.

### Coalition outcomes

#### Coalition partner selection

The one-stage IPD meta-analysis to examine the effects of perspective-taking on partner selection included from *N* = 2,340, subjects in *K* = 5 studies. The model accounted for 38% of the total variance (Pseudo-*R*^2^ = .38), with level-1 ICC_subjects_ = 0.38, and level-2 ICC_experiments_ = 0.00, indicating that most variance was explained by heterogeneity between participants within experiments, and not by variation between the experiments.

**Figure 7.**
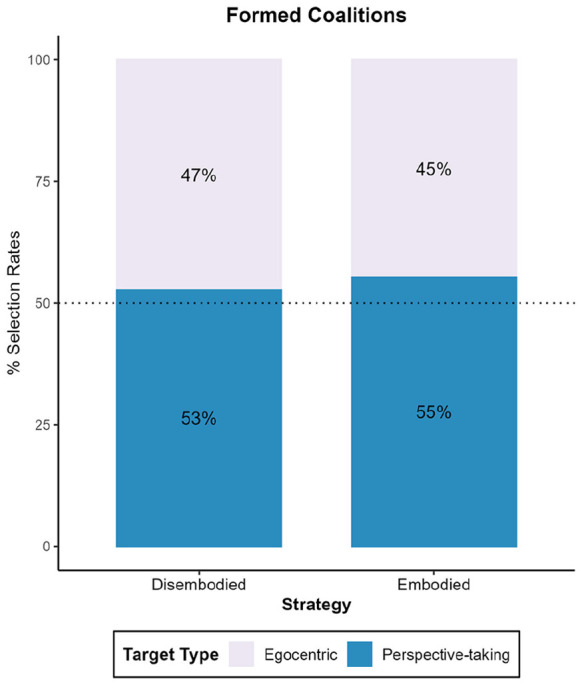
Partner selection rates across Experiments 1 to 4. *Note.* This graph depicts the percentage of times both embodiers and disembodiers chose the coalition partner whose perspective was adopted during the VSPT task (perspective-taking target) versus the coalition partner whose perspective was not adopted (egocentric target). VSPT = visuo-spatial perspective-taking.

The model’s intercept (*OR* = 0.53, 95% CI [0.49, 0.57], *t*(2,338) = 27.69, *p* < .001) revealed that overall perspective-taking influenced coalition partner selection, as participants preferred to form a coalition with the perspective-taking target (54.4%, *n* = 1,275) over the egocentric target (45.6%, *n* = 1,068). Consistent with our predictions, these results, depicted in Figure 7, showed that engaging in perspective-taking with a potential coalition partner increased the preference for this partner over another for whom perspective was not considered. However, contrary to our expectations, the specific strategy participants used during the perspective-taking induction (embodied vs. disembodied) did not influence partner selection in this context, *OR* = 0.03, 95% CI [−0.02, 0.07], *t*(2,338) = 1.14, *p* = .254. Specifically, the rates of choosing the perspective-taking versus egocentric targets were comparably high for embodiers (55.2%, *n* = 876) and disembodiers (52.7%, *n* = 399).

### Perspective-taking outcomes

#### Liking and similarity ratings

The one-stage IPD meta-analysis examining the impact of perspective-taking on liking rates included data from *N* = 2,347 subjects across *k* = 5 studies. The model accounted for 55% of the total variance (Pseudo-*R*^2^ = .55), with level-1 ICC_subjects_ = 0.54, and level-2 ICC_experiments_ = 0.01, indicating most clustering between subjects within experiments, and negligible variance was attributable to differences between the experiments.

**Figure 8.**
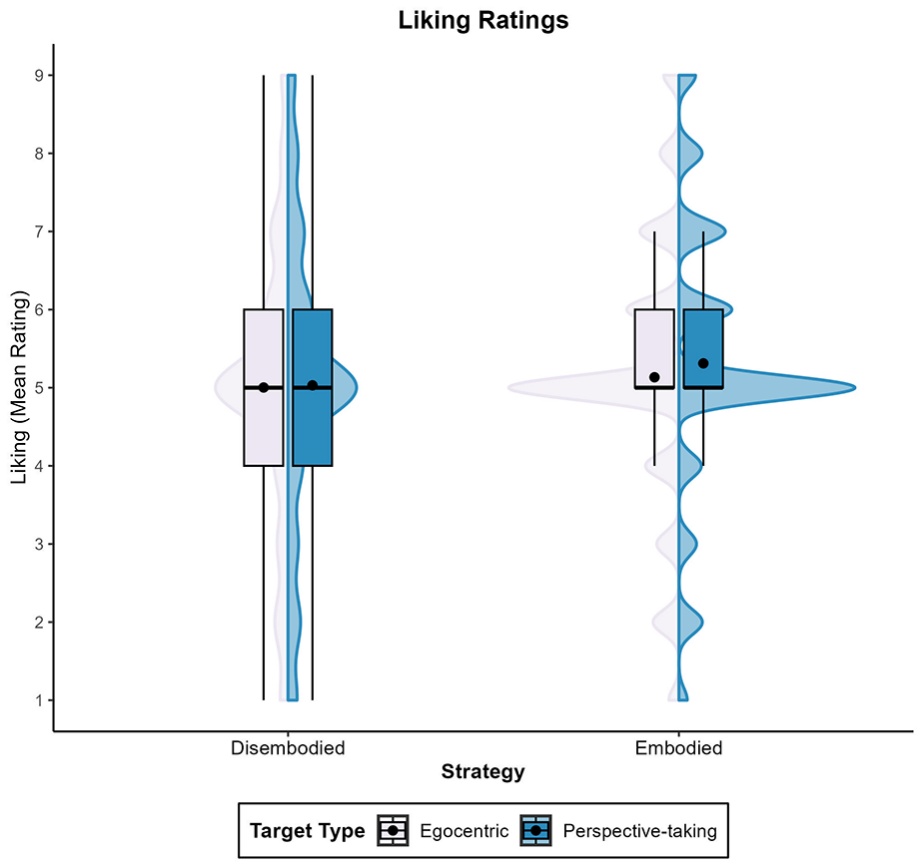
Mean liking ratings across Experiments 1 to 4. *Note.* This graph shows the mean liking ratings for the perspective-taking and egocentric targets, based on the strategy (embodied vs. disembodied) used during the VSPT task. VSPT = visuo-spatial perspective-taking.

The effects of both strategy, β = .14, 95% CI [−0.01, 0.29], *t*(4,679) = 1.79, *p* = .074, and target type, β = .03, 95% CI [−0.09, 0.15], *t*(4,679) = 0.46, *p* = .648, were not significant. Moreover, we found an interaction effect between target type and strategy, β = .15, 95% CI [0.01, 0.29], *t*(4,679) = 2.03, *p* = .042, suggesting that the strategy used modulated the influence of perspective-taking on liking ratings. Results of post-hoc tests, as illustrated in Figure 8, showed that embodiers reported higher levels of liking toward the perspective-taking target relative to the egocentric one, β = .18, 95% CI [0.09, 0.26], *t*(3,167) = 4.20, *p* < .001, whereas disembodiers showed no differences in liking between the two targets, β = .03, 95% CI [−0.09, 0.14], *t*(1,509) = 0.47, *p* = .637. Moreover, the perspective-taking target received higher ratings from embodiers (*M* = 4.31, *SD* = 1.70), than from disembodiers (*M* = 4.03, *SD* = 1.87), β = .28, 95% CI [0.12, 0.43], *t*(2,338) = 3.56, *p* < .001. In contrast, no significant differences were observed in the liking levels toward the egocentric target between embodiers (*M* = 4.13, *SD* = 1.70) and disembodiers (*M* = 4.00, *SD* = 1.80), β = .13, 95% CI [−0.02, 0.28], *t*(2,338) = 1.65, *p* = .100.

Regarding the effects of perspective-taking on similarity rates, the one-stage IPD meta-analysis included data from *N* = 2,340 subjects across *k* = 5 studies. The model accounted for 64% of the total variance (Pseudo-*R*^2^ = .64), with level-1 ICC_subjects_ = 0.63, and level-2 ICC_experiments_ = 0.01, indicating that variance can be mostly attributed to heterogeneity between subjects within experiments, rather than between experimental conditions.

**Figure 9.**
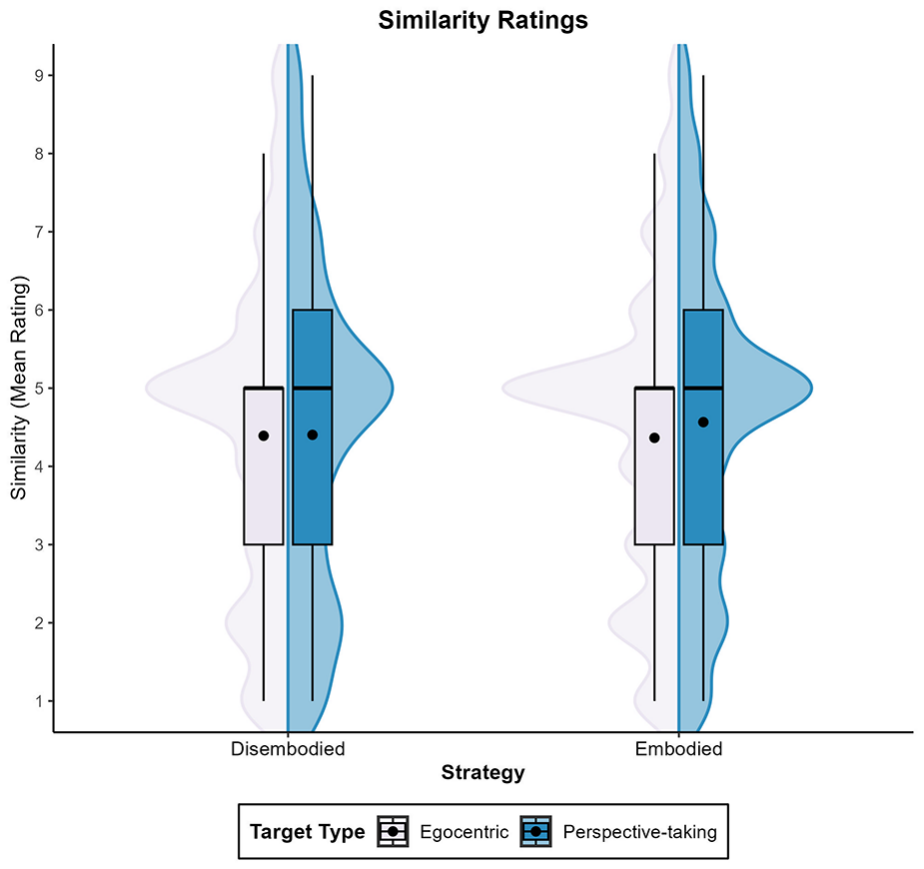
Mean similarity ratings across Experiments 1 to 4. *Note*. This graph shows the mean perceived similarity ratings for the perspective-taking and egocentric targets, based on the strategy (embodied vs. disembodied) used during the VSPT task. VSPT = visuo-spatial perspective-taking.

The effects of target type, β = .01, 95% CI [−0.11, 0.13], *t*(4,679) = 0.19, *p* = .847, and strategy, β = −.03, 95% CI [−0.20, 0.15], *t*(4,679) = −0.30, *p* = .765, alone did not influence perceived similarity. Importantly, we found an interaction between target type and strategy, β = .19, 95% CI [0.04, 0.34], *t*(4,679) = 2.52, *p* = .012. Results of post-hoc tests (Figure 9), showed that embodiers reported higher perceived similarity towards the partner whose perspective they had taken (*M* = 3.57, *SD* = 1.97), relative to the egocentric partner (*M* = 3.36, *SD* = 1.93), β = .20, 95% CI [0.12, 0.29], *t*(3,167) = 4.69, *p* < .001. In contrast, disembodiers did not show differences in perceived similarity between partners (Perspective-taking: *M* = 3.40, *SD* = 2.09; Egocentric: *M* = 3.39, *SD* = 2.06), β = .01, 95% CI [−0.11, 0.13], *t*(1,509) = 0.20, *p* = .845. As expected, there were no differences in reported similarity towards the egocentric target between embodiers and disembodiers, β = −.03, 95% CI [−0.20, 0.14], *t*(2,341) = −0.31, *p* = .760. Additionally, contrary to our predictions, embodiers did not report higher perceived similarity towards the perspective-taking target than disembodiers, β = .16, 95% CI [−0.01, 0.34], *t*(2,338) = 1.83, *p* = .068.

### Discussion

Aggregating all our data gives us the clearest indication of how participants performed on the VSPT task. The internal meta-analysis showed that participants took longer and made more errors when taking someone else’s perspective. However, we also found that the strategy used during the perspective-taking induction impacted their performance, as disembodiers were faster but less accurate than embodiers, and were more accurate when responding from an egocentric perspective than when taking others. This pattern of results aligns with Kessler and Wang’s (2012) findings. Their explanations suggest that embodiers might be slower because they more deeply embody others’ perspectives, leading to higher accuracy but increased processing time. Conversely, disembodiers might be less accurate because they switch to alternative strategies (e.g., object rotation or rule-based approaches) that prioritize speed over depth of perspective-taking.

Regarding our main hypothesis, we found that perspective-taking influenced coalition partner selection in settings lacking cooperative cues. Adopting the perspective of a potential coalition partner increased the likelihood of selecting this partner over a potential partner whose perspective was not adopted. However, contrary to our expectations, this pattern was not particularly pronounced for individuals who embodied their partners when engaging in perspective-taking.

As for the influence of perspective-taking on social judgments, we found that embodiers showed greater liking and perceived similarity towards the perspective-taking target compared to the egocentric target, while disembodiers showed no difference between targets. For the perspective-taking target specifically, embodiers gave higher ratings than disembodiers. However, ratings for the egocentric target did not differ between embodiers and disembodiers. These results suggest that embodied perspective-taking selectively enhances liking and perceived similarity towards the specific partner whose perspective was adopted.

Considering all measures, we observe that strategy affects both VSPT task performance (accuracy and reaction times) and feelings toward the task targets. Disembodiers were faster but made significantly more errors during perspective-taking trials. This suggests they may indeed follow a geometric mirroring strategy but sometimes neglect the final step, indicating a speed-accuracy trade-off. Consequently, they perform particularly poorly on perspective-taking target trials, which aligns with their lower ratings of liking and similarity for the perspective-taking target compared to embodiers.

Intriguingly, despite these differences in task performance and social judgments, both embodiers and disembodiers showed similar decisions when forming a coalition (main effect of perspective-taking). This discrepancy between social judgments and behavioral outcomes will be further discussed in the following section.

## General discussion

Our research examined the influence of VSPT on coalition partner selection in scenarios where potential partners are indistinguishable a priori, and where major coalition theories would therefore predict random selection. While the individual results across our experiments were inconsistent, a meta-analysis suggested that perspective-taking exerts a modest influence on partner selection. However, the hypothesized self-other merging mechanism, which we posited as the driver of this effect, received only partial support.

Our main research question sought to determine whether perspective-taking influences coalition partner selection even when objective cues typically guiding coalition formation are absent. We found that when potential partners are a priori not distinguishable, participants showed a tendency to form coalitions with partners whose perspective they had adopted (perspective-taking target) over those they had not (egocentric target). This effect, while small, is practically significant in coalition contexts, representing approximately a 10% swing in inclusion rates for the perspective-taking target (54:45). Such a margin could prove decisive in many real-world coalition scenarios, offering an explanation for partner selection in situations where major theories of coalition formation would predict random selection.

The mechanisms driving this effect were not fully elucidated, but our findings provide important insights. The embodiment hypothesis, which suggests that perspective-taking effects arise from an embodied simulation of physical closeness (e.g., Erle & Topolinski, 2017), received support among individuals who engaged in embodied simulation. Embodiers perceived greater social closeness to the perspective-taking target, reporting higher liking and similarity ratings, and they selected this target more often as a coalition partner. This pattern aligns with our expectations and reinforces the role of self-other merging as a mechanism underlying perspective-taking effects in individuals who employ embodied strategies. However, disembodiers presented a more complex and unexpected pattern. As anticipated, they did not report increased social closeness toward the perspective-taking target. Yet, contrary to our predictions, they still exhibited a preference for this target in coalition formation. This suggests that while embodied simulation could explain perspective-taking effects for embodiers, other mechanisms must be driving these effects in disembodied individuals.

Interestingly, embodiers were the most consistent group across our studies. Their performance during the perspective-taking induction aligned with expectations and mirrored the pattern reported by Kessler and Wang (2012). They also showed increased liking and similarity ratings towards the perspective-taking target, and selected it more often as a coalition partner. This consistent pattern suggests that for embodiers, both their judgments and decisions could indeed be driven by self-other merging through embodied simulation.

In contrast, disembodiers presented greater variability across experiments. Their performance during perspective-taking tasks was inconsistent, their social judgments showed no clear preference, or even favored the egocentric target in some cases, yet they mostly opted for the perspective-taking target in coalition formation. This inconsistency may have weakened the overall relationship between embodied perspective-taking and coalition decisions that we had expected to observe, but crucially, it raises questions about the mechanisms driving their decisions.

Disembodiers’ behavior suggests that mechanisms other than embodied merging underlie their coalition preferences. One possibility is that the greater cognitive effort required for perspective-taking trials led them to pay increased attention to the perspective-taking target, making it more salient and familiar. This attentional effect could influence partner selection independently of social closeness, which aligns with our finding that disembodiers performed worse on perspective-taking trials and only showed only effects on partner selection despite not showing increased liking or similarity in their social judgments. Moreover, we reason that their variability across experiments is perhaps not surprising, as disembodied individuals may use different disembodied strategies, such as abstract rule-following, object rotation, or other approaches, which could involve differential perspective-taking effects.

Another stream of findings worth mentioning relates to the role of (visuo-spatial) perspective-taking on more economic variables, such as money allocation and reservation price. Contrary to our expectations and prior findings (Cantiani, van Beest, Cruijssen, et al., 2024; Cantiani et al., 2025), none of the experiments found that perspective-taking increased the amount of money allocated to coalition partners or led participants to reduce their reservation price (these analyses are reported in the Supplemental Material). In hindsight, this may have been an unrealistic expectation. While perspective-taking may increase liking and/or perceptions of similarity toward an unknown target, this alone may be insufficient to alter allocation preferences, especially in a hypothetical scenario with no real consequences. This may be due to the limitations of hypothetical scenarios without real consequences. While perspective-taking can increase liking and perceptions of similarity, these effects may be too modest to influence economic decisions. Additionally, the 2(1–1–1) game, which elicits a strong preference for equal allocation, unlike the 5(4–3–2) game, where multiple norms apply, may explain the adherence to equality (Schelling, 1978). Finally, the symmetric nature of the game provided insufficient cues for mental state inference, likely preventing changes in economic behavior.

### Limitations and future research directions

Our research combined the statistical strength of meta-analyses with the detailed insights from individual experiments. Each experiment revealed unexpected complexities in studying perspective-taking strategies and their effects in social judgments and decision-making, pointing to the need for more refined task designs and experimental conditions (Quesque et al., 2024). Rather than a limitation, the variability in results across studies offers valuable insights into the complexity of perspective-taking processes and the methodological challenges involved.

One challenge lies in how we assess embodiment in perspective-taking. The discrepancy found between the role of strategy in decisions and judgments could stem from the low precision of self-report measures in our strategy assessment. While self-reports are valuable for accessing subjective experiences, participants may lack the meta-cognitive abilities or awareness needed to accurately report their actions during the task (Ackerman & Thomson, 2017) or may be unwilling to admit perceived “errors” in their performance. For instance, some confused but actual embodiers could have been “refined” into the disembodiers group because they indicated using both strategies. However, in favor of the validity of our current assessment, our self-reported strategies produced VSPT performance patterns previously observed when embodiment was manipulated through body posture. Specifically, embodiers demonstrated increased accuracy but longer reaction times, consistent with prior findings (Kessler & Wang, 2012). This alignment supports the idea that our classification captures meaningful differences in perspective-taking strategies. Nonetheless, developing more precise and refined methods of assessing strategies in VSPT tasks is a current demand in the field (Quesque et al., 2024). Future research could (a) further investigate when and how *embodiers* and *disembodiers* produce comparable effects despite relying on different perspective-taking routes, (b) whether, if so, how using different disembodied strategies leads to differential perspective-taking effects, and (c) incorporate objective measures to complement self-reports. For example, by incorporating response time patterns paired with posture congruency manipulations, similar to the approach used by Kessler and Rutherford (2010), could aid in classification. Furthermore, peripheral and brain measures, which have previously provided evidence of the embodied nature of VSPT (Gooding-Williams et al., 2017; Gunia et al., 2024; Martin et al., 2020), could also help assess and distinguish between embodiers and disembodiers.

A related limitation concerns the use of action-oriented instructions in our VSPT task. Asking participants to “grab” an object may have implicitly triggered embodied processing even in those who reported using disembodied strategies, as prior research has shown (Tversky & Hard, 2009). This could partly explain why both groups showed sensitivity to perspective-taking. While it is possible that removing the action-oriented working might reduce embodiment overall, we do not believe this accounts for the differences between participants who reported using embodied strategies and those who did not. More critically, changing the action-oriented instruction into a visual-oriented instruction, would not address our central finding: those who did not report using embodied strategies, and did not show greater liking or similarity, still preferred the perspective-taking partner. If the instruction had induced comparable embodiment across participants, we would expect comparable social judgments across groups.

We also found that both the decision options and format were more influential in coalition formation than anticipated, which may explain the observed discrepancy between social judgments and decisions. Regarding decision options, when participants had the option to form a coalition with both partners (a grand coalition), the preference for the perspective-taking target disappeared, unlike in scenarios where they were forced to choose only one partner. While we speculate that these results could be due to perspective-taking generally promoting greater inclusion, as seen in previous research (Cantiani, van Beest, Cruijssen, et al., 2024; Cantiani et al., 2025), or that the lack of differences was due to sample size limitations, it also suggests that the study design influenced participants’ responses, potentially contributing to the discrepancies we observed between their social judgments and coalition behavior.

Regarding the decision format, we uncovered a pervasive laterality bias mostly consistent with the *body-specificity hypothesis* (Casasanto, 2009; Casasanto & Chrysikou, 2011. This bias affected partner selection when participants were presented with a dichotomous response format. This hypothesis posits that our physical characteristics—such as handedness—affect how we think, feel, and make decisions, leading individuals to associate positive concepts with their dominant side and negative concepts with their non-dominant side. In our study, the majority of participants (84% of whom were right-handed) preferred the target located on the right side of the screen. While this bias did not fully explain our results, it had a substantial impact on outcomes (Experiment 3, *OR* = 0.48). This laterality effect, though not part of coalition theories, was nearly as strong as our predicted effect for coalition partner selection (IPD, *OR* = 0.53). Although this laterality bias is not and should not be embedded within coalition formation theories, it underscores that non-economic factors, such as visual positioning, can exert substantial effects on decision-making behaviors.

A related methodological consideration involves order effects: moving the social ratings phase from before the coalition decision stage in Experiment 1 to after it in Experiment 2 onward eliminated the effects initially observed in Experiment 1. In hindsight, this could suggest that reflection might be necessary to amplify the influence of perspective-taking on decisions. Together, these findings stress the importance of also considering individual studies and their methodological challenges. Addressing these issues in future research will be essential for refining our understanding of perspective-taking processes.

### Theoretical and practical implications

From a broader perspective, our findings contribute to the growing body of evidence supporting the importance of psychological factors in coalition formation, as proposed by previous research (van Beest et al., 2011). Recent findings have demonstrated that the disposition to take others’ perspectives enhances coalition formation negotiations in 5(4–3–2) weighted majority games by utilizing objective cues to infer the likely behavior of potential partners and strategically adapting to them. In contrast, our findings show that perspective-taking exerts influence on coalition formation even in the absence of such cues, as demonstrated in the 2(1–1–1) game. In this setting, where all players hold equal resources and no objective reasons exist to prefer one partner over another, coalition theories predict random partner selection. However, our results reveal that a purely psychological manipulation of perspective-taking, devoid of economic context, can also influence coalition behavior. This underscores the profound impact of cognitive and perceptual processes on coalition dynamics, highlighting that these processes can drive coalition preferences even in the absence of clear rational or economic motivations.

We deliberately employed the 2(1–1–1) game, where participants are equal on all possible dimensions, as it provided a better test of our hypothesis. In such a context, there is no rational basis for preferring one coalition over another, so any observable preference must be attributed to the manipulation of perspective-taking. Future research could examine whether VSPT also influences partner selection and economic offers in more complex games like the 5(4–3–2) weighted majority game. In such scenarios, established coalition formation theories make specific predictions about how objective cues determine preferences, and perspective-taking may use these cues to infer intentions and behaviors. This approach would help clarify the interplay between psychological factors and rational economic considerations in coalition formation across various contexts.

These findings have practical implications for individuals and organizations involved in coalition formation efforts. Major coalition formation theories that rely solely on objective cues may predict random partner selection in many real-life situations, where cues are often subtle, ambiguous, and heavily influenced by subjective perceptions. By considering psychological processes such as perspective-taking, we can better predict partner preferences and explain outcomes that might otherwise seem inexplicable. Understanding these subtle psychological influences on partner selection can inform strategies for more effective coalition formation and maintenance, particularly when potential partners are not easily distinguishable based on objective criteria.

Moreover, our findings regarding the discrepant role of perspective-taking strategies on social judgments and partner selection have four key implications for the perspective-taking literature. First, we extend previous research, which primarily examined the social effects of perspective-taking through psychological inductions (e.g., vignettes or written instructions prompting individuals to imagine others’ thoughts or feelings), by showing that VSPT, where individuals literally adopt another’s viewpoint, can also influence social judgments, such as increased liking and perceived similarity. Furthermore, we showed that this effect occurs in a group setting, where individuals make partner selection decisions, addressing variables and contexts that previous research has not yet examined. Second, it supports the hypothesis that social closeness induced through perspective-taking is more pronounced among those who effectively simulate taking another person’s perspective, but not among individuals who use other strategies that do not require embodied perspective-taking. This finding contributes to ongoing debates in the field (Cole et al., 2020; Quesque et al., 2024) by examining the overlooked role of strategy in social judgments and emphasizing the need to consider this aspect in future studies. Third, it showed that the strategy impacted how individuals perceived and felt toward others, even if it did not directly influence partner selection. Last, it challenges the assumption that self-other merging through embodied simulation is the primary factor driving coalition preferences across all participants. While this mechanism may explain the behavior of embodiers, it fails to account for the choices made by disembodiers. This discrepancy evidence the need for further research to understand why disembodiers prefer the perspective-taking target in coalition formation despite not showing increased social closeness, and to explore potential explanations for their inconsistent behavior across studies. Participants’ self-reports about their perspective-taking strategies evidence the inter-individual differences we inevitably encounter in VSPT research. Our findings make clear that simplified, single-process theories of VSPT are unlikely to capture how people actually take others’ perspectives.

## Conclusion

In conclusion, our results suggest that taking another’s (visuo-spatial) perspective can influence social connections and coalition formation, even when potential partners are indistinguishable. This research contributes to our understanding of the psychological factors shaping coalition formation in scenarios where existing theories predict random partner selection. Additionally, our results provide insights into the mechanisms behind these effects. While we found support for the embodiment hypothesis, showing that engaging in embodied simulation during perspective-taking fosters social closeness and, in turn, influences partner selection, our findings also reveal that perspective-taking effects extend beyond embodied strategies. Even individuals using disembodied strategies still demonstrated a preference for their perspective-taking target, despite no measurable differences in perceptions of social closeness. This suggests that additional mechanisms, beyond embodied simulation, contribute to the link between perspective-taking and coalition formation.

## Supplemental Material

sj-docx-1-qjp-10.1177_17470218251358231 – Supplemental material for Now that I see it your way, I choose you: Visuo-spatial perspective-taking affects partner selection during coalition formation Supplemental material, sj-docx-1-qjp-10.1177_17470218251358231 for Now that I see it your way, I choose you: Visuo-spatial perspective-taking affects partner selection during coalition formation by Anabela Cantiani, Ilja van Beest and Thorsten M Erle in Quarterly Journal of Experimental Psychology
