# Supplementary material for: Now that I see it your way, I choose you: Visuo-spatial perspective-taking affects partner selection during coalition formation
Source: Q J Exp Psychol (Hove). 2025 Jul 8;79(4):841–66. doi: 10.1177/17470218251358231 (PMC12982561; doi:10.1177/17470218251358231)
Supplement: sj-docx-1-qjp-10.1177_17470218251358231 – Supplemental material for Now that I see it your way, I choose you: Visuo-spatial perspective-taking affects partner selection during coalition formation [file sj-docx-1-qjp-10.1177_17470218251358231.docx]

Supplementary Material for:

# Now That I See it Your Way, I Choose You: Visuo-Spatial Perspective-Taking Affects Partner Selection During Coalition Formation

**Author, email, orcid:**

1. Anabela Cantiani^a^, [A.A.Cantiani@tilburguniversity.edu](mailto:A.A.Cantiani@tilburguniversity.edu), 0009-0003-4611-6957
2. Ilja van Beest^a^, [I.VanBeest@tilburguniversity.edu](mailto:I.VanBeest@tilburguniversity.edu), 0000-0003-2855-3638
3. Thorsten M. Erle^a^, [T.M.Erle@tilburguniversity.edu](mailto:T.M.Erle@tilburguniversity.edu), [0000-0003-3477-5106](https://orcid.org/0000-0003-3477-5106)

^a^ Department of Social Psychology

Tilburg School of Social and Behavioral Sciences

Tilburg University

Postbus 90153, 5000 LE, The Netherlands

**Corresponding author:**

Anabela Cantiani; Tilburg University, [A.A.Cantiani@tilburguniversity.edu](mailto:A.A.Cantiani@tilburguniversity.edu)

# Supplementary Material: Measured Variables and Additional Analyses

This supplementary material presents additional analyses that we have carried out and that have not been included in the main text, as well as further details regarding additional variables that were measured for exploratory purposes.

# Additional Analyses

## Laterality Effects in Coalition Partner Selection

We observed conflicting behaviors in Experiment 1 and Experiment 2, particularly in how the disembodiers (participants who do not use an embodied strategy while engaging in perspective-taking) responded. In Experiment 1, disembodiers preferred the egocentric target, while in Experiment 2, their choices were more similar to the embodiers (participants who employ an embodied strategy while perspective-taking), showing a slight, but not significant, preference for the perspective-taking target. This prompted us to review the results of Experiment 1 more closely.

We examined whether the position of the two targets (egocentric and perspective-taking) influenced participants' choices in Experiment 1. In the analysis, we considered two positions: Position 1, where the perspective-taking target was on the right, and Position 2, where the egocentric target was on the right.

The results showed that target position had a significant impact. Participants were much less likely to choose the perspective-taking target when the egocentric target was on the right (Position 2), *OR* = 0.25, *95%CI* [0.09, 0.71], *p* = .010. However, the overall strategy participants used (egocentric or perspective-taking) did not significantly affect their choices, *OR* = 2.40, *95%CI* [0.95, 6.11], *p* = .065, and there was no significant interaction between target position and strategy, *OR* = 0.89, *95%CI* [0.25, 3.30], *p* = .859. The non-significant intercept, *OR* = 1.31, *95%CI* [0.64, 2.75], *p* = .467, indicates no overall preference for one target or strategy over the other, independent of target position.

## Money Allocation

### Experiment 1

The overall model was not statistically significant (*F*(3, 189) = 0.32, *p* = .808), and the adjusted R^2^ indicated a poor fit of the model to the data (*adjusted R^2^* = -0.01). Neither the target type, β = -0.48, *95%CI* [-1.96, 1.01], *t*(189) = -0.64, *p* = .526, nor the strategy used to solve the VPT task, β = -0.48, *95%CI* [-1.72, 0.75], *t*(189) = -0.77, *p* = .444, nor the interaction between these variables, β = 0.40, *95%CI* [-1.42, 2.23], *t*(189) = 0.44, *p* = .662, affected monetary offers.

### Experiment 2

The overall model was not statistically significant (*F*(1, 221) = 1.23, *p* = .269) and the adjusted R^2^ indicated a poor fit of the model to the data (*adjusted R^2^* = 0.01). Neither the target type, β = -1.58, *95%CI* [-3.20, 0.03], *t*(219) = -1.93, *p* = .055, nor the strategy, β = 0.09, *95%CI* [-1.34, 1.51], *t*(219) = 0.12, *p* = .906, nor their interaction, β = 1.50, *95%CI* [-0.46, 3.46], *t*(219) = 1.51, *p* = .132, affected monetary offers.

### Experiment 3

The overall model was not statistically significant (*F*(3, 935) = 2.02, *p* = .110) and the adjusted R^2^ indicated a poor fit of the model to the data (*adjusted R^2^* < 0.01). The strategy used to solve the VPT task showed a significant effect on offer sizes, β = 0.72, *95%CI* [0.06, 1.38], *t*(935) = 2.14, *p* = .033, with embodiers allocating more money to their partners (*M* = 10.9, *SD* = 3.64) than disembodiers (*M* = 10.4, *SD* = 3.21). Neither the target type, β = 0.09, *95%CI* [-0.64, 0.82], *t*(935) = 0.25, *p* = .805, nor the interaction term significantly affect offers, β = -0.41, *95%CI* [-1.32, 0.49], *t*(935) = -0.90, *p* = .369.

### Experiment 4a

The overall model was not statistically significant (*F*(3, 485) = 0.03, *p* = .991) and the adjusted R^2^ indicated a poor fit of the model to the data (*adjusted R^2^* < 0.01). Neither the target type, β = -0.06, *95%CI* [-1.28, 1.16], *t*(485) = -0.09, *p* = .927, northe strategy, β = 0.09, *95%CI* [-1.06, 1.24], *t*(485) = 0.16, *p* = .875, nor the interaction term, β = -0.00, *95%CI* [-1.45, 1.45], *t*(485) = -0.00, *p* = .999, affected offers.

### Experiment 4b

The overall model was not statistically significant (*F*(3, 494) = 0.68, *p* = .562) and the adjusted R^2^ indicated a poor fit of the model to the data (*adjusted R^2^* < 0.01). Neither the effects of target type, β = -0.55, *95%CI* [-1.72, 0.61], *t*(494) = -0.93, *p* = .352, nor strategy, β = -0.26, *95%CI* [-1.24, 0.71], *t*(494) = -0.53, *p* = .598, nor the interaction term, β = 0.20, *95%CI* [-1.18, 1.58], *t*(494) = 0.28, *p* = .780, was significant.

## Reservation Price

### Experiment 1

The overall model was not statistically significant, *F*(3, 189) = 0.54, *p* = .652, indicating that the predictors did not explain a significant portion of the variance in reservation price, and the adjusted R^2^ indicated a poor fit of the model to the data (*adjusted R^2^* = -0.01). Specifically, neither the interaction between selected partner and strategy, β = -0.56, *95%CI* [-2.66, 1.55], *p* = .603, nor their main effects of selected partner, β = 0.68, *95%CI* [-1.04, 2.39], *p* = .437, or strategy, β = -0.32, *95%CI* [-1.75, 1.10], *p* = .654, were significant predictors of reservation price.

### Experiment 2

The overall model was not statistically significant, *F*(3, 218) = 0.18, *p* = .911, indicating that the predictors did not explain a significant portion of the variance in reservation price, and the adjusted R^2^ indicated a poor fit of the model to the data (*adjusted R^2^* = 0.01). Specifically, neither the interaction between selected partner and strategy, β = -0.26, *95%CI* [-1.95, 1.42], *p* = .759, nor their main effects of selected partner, β = 0.41, *95%CI* [-0.98, 1.80], *p* = .564, or strategy, β = 0.27, *95%CI* [-0.96, 1.50], *p* = .665, were significant predictors of reservation price.

# Measured Variables

In this section, we report the full set of variables included in the experiments, along with the specific order in which they were measured. We also detail the exploratory variables included in the experiments that were not described in the methods sections of the main manuscript.

## Experiment 1

Experiment 1 included a Visual Perspective-Taking (VPT) task consisting of 16 trials, followed by sympathy and similarity ratings. Next, participants completed a coalition partner selection task using a dichotomous format. Following this, they performed a money allocation task in which they made offers and indicated their reservation price, defined as the minimum amount of money they were willing to accept from a partner to form a coalition. The experiment concluded with a strategy assessment. An additional variable, altercentric intrusion, was included for exploratory purposes. This measured the extent to which participants felt their accuracy and speed during the VPT task were influenced by another person's perspective, using a Likert scale from 1 ("Not at all") to 10 ("Extremely").

## Experiment 2

**Experiment 2 followed a structure similar to Experiment 1, starting with a 16-trial VPT task. Participants then completed a coalition partner selection task, which, unlike Experiment 1, included the option to form grand coalitions. Following this, they participated in the money allocation task, which involved making offers and specifying reservation prices, and provided ratings for sympathy and similarity. A refined strategy assessment was also included in this experiment.**

**The exploratory variables in Experiment 2 were: (a) Social Value Orientation (Murphy et al., 2011) – a measure of concern for others, where participants chose between different money distribution options for themselves and an unknown person; (b) The altercentric intrusion measure from Experiment 1; and (c) An open-ended question about the goal of the study.**

## Experiment 3

**Experiment 3** involved a 16-trial VPT task, followed by participants expressing their intentions to form coalitions on a Likert scale. They then completed a coalition partner selection task using the dichotomous format and engaged in a money allocation task (offers only). As in the previous experiments, they provided sympathy and similarity ratings and completed a refined strategy assessment.

In this experiment, handedness was included as an exploratory variable. Participants were asked, "*Please indicate your handedness*" with the following response options: "*Right-handed*", "*Left-handed*", or "*Ambidextrous*".

## Experiment 4a

**Experiment 4a** expanded the number of VPT trials to 32. Participants rated their intentions to form coalitions on a Likert scale, followed by a coalition partner selection task using a non-dichotomous format. Afterward, they completed the money allocation task, provided sympathy and similarity ratings, and completed the refined strategy assessment. Handedness was again included as an exploratory variable.

## Experiment 4b

Experiment 4b followed the same structure as Experiment 4a but implemented a block design.
